# Supplementary material for: Microplastics affect sedimentary microbial communities and nitrogen cycling
Source: Nat Commun. 2020 May 12;11:2372. doi: 10.1038/s41467-020-16235-3 (PMC7217880; doi:10.1038/s41467-020-16235-3)
Supplement: Supplementary file 1 — Supplementary Information [file 41467_2020_16235_MOESM1_ESM.pdf]

Supplementary information for Microplastics affect sedimentary microbial communities and nitrogen cycling by Meredith E. Seeley*, Bongkeun Song, Renia Passie and Robert C. Hale

SUPPLEMENTARY METHODS

Flame retardant and phthalate additives were determined in the polymers PE, PVC and PUF. Penta-BDE is a (poly)brominated diphenyl ether (BDE) mixture consisting of several major congeners: BDE47, 99, 100, 153 and 154[1]. Penta-BDE was commonly used in PUF. Components of Firemaster® 550, a replacement flame retardant for Penta-BDE, were also determined (i.e. 2-ethylhexyl 2, 3, 4, 5-tetrabromobenzoate (TBB); 2-ethylhexyl 2, 3, 4, 5-tetrabromophthalate (TBPH)) and several phosphate FRs: tris (1,3-dichloro-2-propyl) phosphate (TDCPP)) and triphenyl phosphate (TPP)). Tris (2-chloroethyl) phosphate (TCEP) and tris (1-chloro-2-propyl) phosphate (TCPP), additional phosphate-based flame retardants, were also analyzed. Three aliquots of finely milled PE, PVC and PUF (53-300 µm) were chemically analyzed. These were sequentially extracted with three 60 mL aliquots of dichloromethane (DCM). Extract aliquots were combined and reduced in volume (0.5 mL) under a stream of high purity nitrogen. All solvents used were high purity HPLC or residue grade from Burdick & Jackson. Residual solids in the extracts were removed by filtration (Whatman filter paper 11 µm, 7 cm diameter). The solvent extracts were purified to remove high molecular weight interferences by size exclusion liquid chromatography (Envirosep ABC column. 350 x 21.2 mm. Phenomenex Inc.) and then by elution through 2-g solid phase silica gel glass extraction columns (Isolute, International Sorbent Tech.; Hengoed Mid Glamorgan, U.K.)[1]. Silica gel eluents were S1: 3.5ml (100% hexane-waste), S2: 6.5ml (60:40, hexane/methylene chloride-hydrophobic flame retardants) + 8mL (100% methylene chloride) and S3: 5ml (50:50, methylene chloride /acetone-polar compounds). The elution profiles of the additives were determined by processing standards of the target chemicals. Purified extracts were diluted to produce chromatographic peak areas within the bounds of the LC/MS or GC/MS calibration curves.

Identification and quantitation of the target additives from PUF, PE, and PVC were accomplished by ultra-high performance liquid chromatography/mass spectrometry (UHPLC/MS). The UHPLC (Acquity UHPLC, Waters Corporation, Milford, MA, U.S.A.) was operated in the gradient mode and equipped with a C_{18} UPLC analytical column (Acquity UPLC BEH C18, 1.7 μm particle diameter, 2.1 × 150 mm, Waters Corp. or equivalent). The UHPLC column temperature was maintained at 45°C and the mobile phase consisted of 100% water (A1) and 100% methanol (B1). The initial mobile phase composition was 95:5 A1/B1 at a flow rate of 250μL/minute, held for 3 minutes. This was followed by a linear gradient to 30:70 A1/B1 over the next 12 minutes. The flow rate was then increased to 300 μL/minute, followed by a 5-minute linear solvent gradient to 100% methanol. The column was regenerated via a 3-minute linear gradient back to 95:5 A1/B1, followed by a 2 min hold at a flow rate of 250 μL/min. Analytes were subjected to atmospheric pressure photoionization (APPI), the dopant (acetone) was introduced at 150 μL/min by a liquid chromatography pump (LC-20AD, Shimadzu Corporation, Kyoto, Japan), and product ions detected on a triple quadrupole mass spectrometer (3200 QTrap, AB Sciex, Framingham, MA, U.S.A.) operated in the multiple reaction monitoring (MRM) mode. The following parameters were used: curtain gas 15 psi (N2), probe temperature 300°C, nebulizer gas 55 psi (Zero air), auxiliary gas 40 psi (Zero air), interface heater on, collision gas medium (N2), ion spray –850 V. TPP in the purified extracts was determined by UPLC-APPI-MS/MS, operated in the positive ion mode (ion spray 850 V). The analytical method has been validated previously for FRs in complex environmental media (La Guardia et al., 2013). Phthalates in PVC were analyzed by GC/MS (Agilent 5975C MS coupled with a 7890A GC). Carrier gas was helium. The GC was equipped with a DB-5 column (Agilent Technologies: 60 m x 0.1 μm film thickness). Analytes were injected in the splitless mode and subjected to

electron impact ionization mode at 70 eV. Ion mass-to-charges were obtained at unit resolution. Compound concentrations were calculated based on the area of selected ions (p-terphenyl m/z 230, DEHP m/z 149 and BDE166 m/z 484).

Of the polymers analyzed, as expected, PUF exhibited the most abundant and diverse additives. It contained both brominated and phosphate flame retardants (Supplementary Table 15). These analytes were not detected in PE and PVC. Phthalate analysis was conducted only for PVC. One phthalate was found, di-ethylhexyl phthalate (DEHP), at a mean concentration of 8.61 mg g^{-1}. Neither phthalates nor flame retardant additives were detected in PE. As the analyses were highly targeted, the presence of other additives in the plastics cannot be excluded.

SUPPLEMENTARY FIGURES

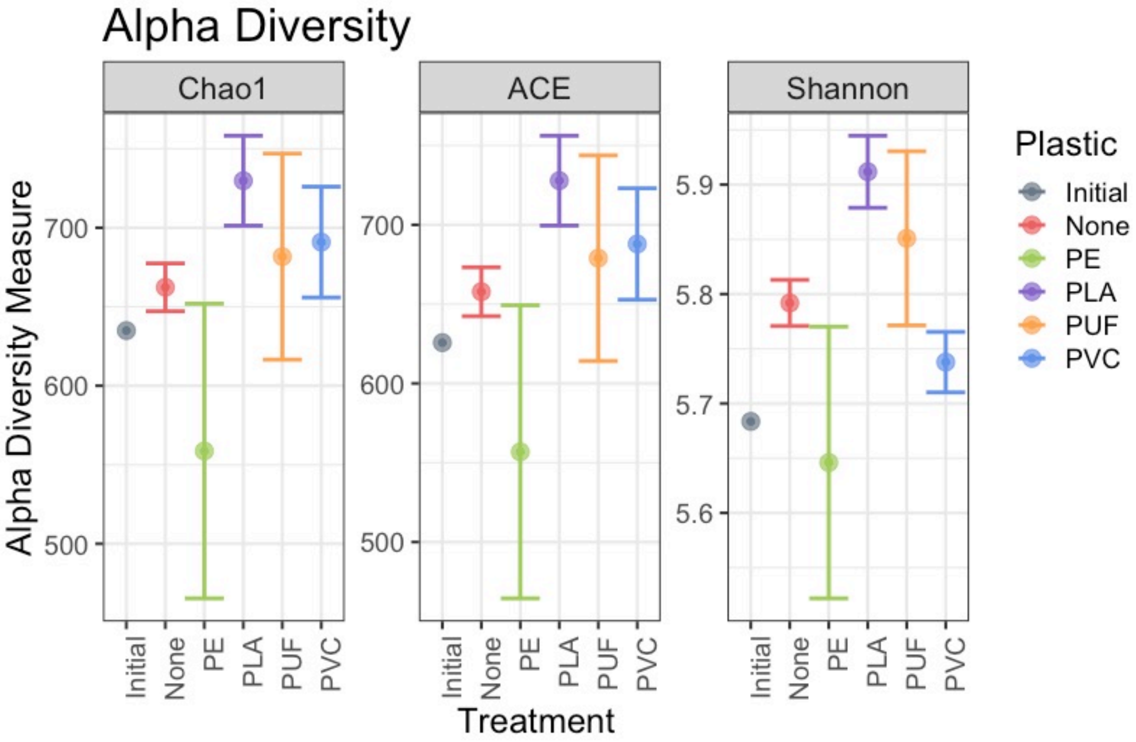

Supplementary Figure 1 Alpha diversity measures for three different diversity statistics, for the average of all 6 samples (3 replicates, 7 and 16 collection days) where error bars represent standard error, with the exception of the initial community, which is a single sample (n = 1).

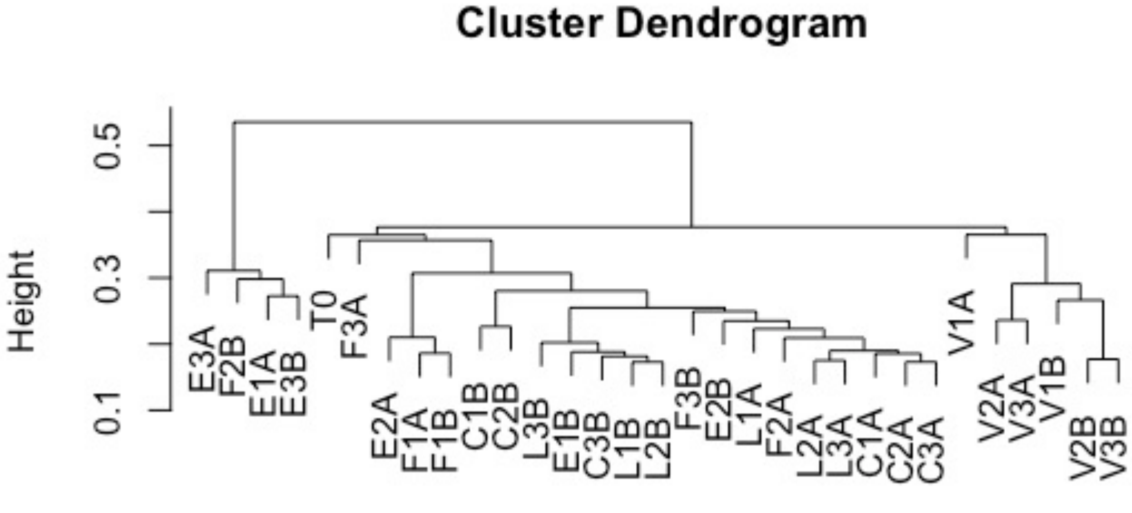

Supplementary Figure 2 Cluster dendrogram generated in phyloseq, illustrating the relatedness of different samples. Samples are named for polymer type (L = PLA, E = PE, F = PUF, V = PVC and C = Control), replicate (1, 2, or 3) and collection date (A = 7 and B = 16 days). T0 is the initial sample. All PVC samples are the most distinctly different lineage, on the right of the dendrogram.

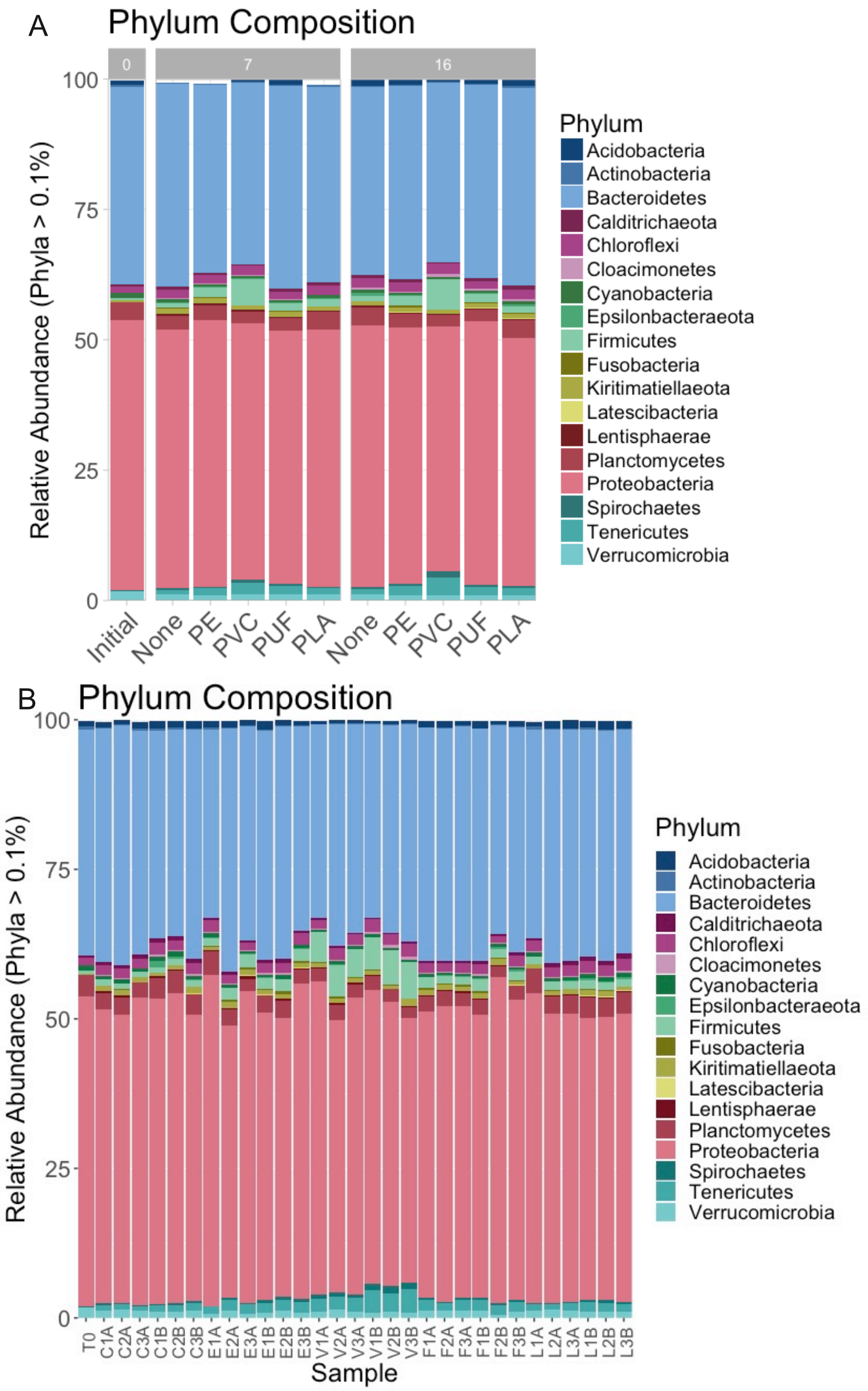

Supplementary Figure 3 Stacked bar plot showing relative abundance for all phyla present in samples at >0.1% relative abundance. In A, samples are averaged (n =3) for each collection date, 7 days or 16 days, and the initial sample is on the left (0 days). For all individual samples (B), samples are named for polymer type (L = PLA, E = PE, F = PUF, V = PVC and C = Control), replicate (1, 2, or 3) and collection date (A = 7 and B = 16 days), and T0 is the initial sample.

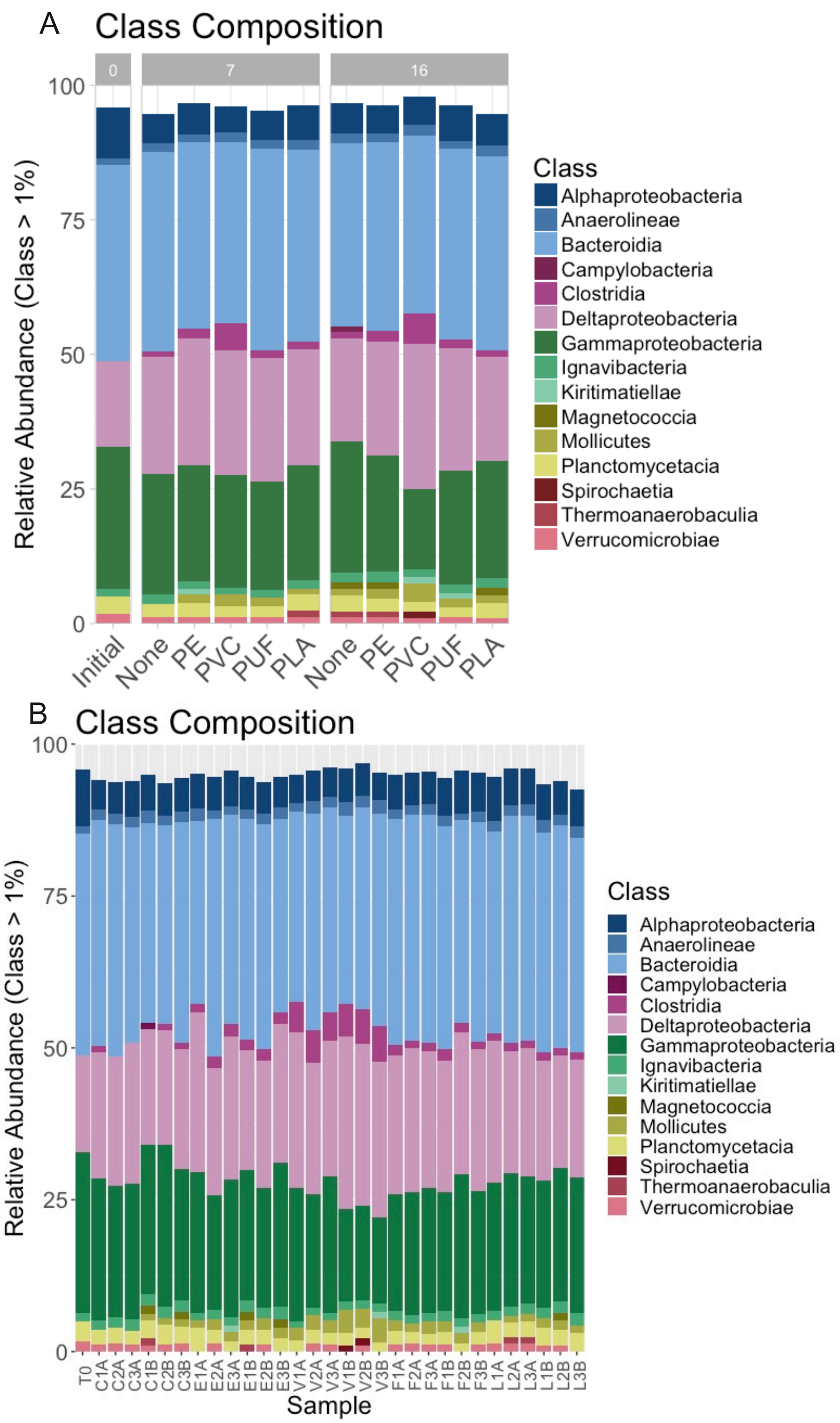

Supplementary Figure 4 Stacked bar plot showing relative abundance for all classes present in samples at >1% relative abundance. In A, samples are averaged (n =3) for each collection date, 7 days or 16 days, and the initial sample is on the left (0 days).). For all individual samples (B), samples are named for polymer type (L = PLA, E = PE, F = PUF, V = PVC and C = Control), replicate (1, 2, or 3) and collection date (A = 7 and B = 16 days), and T0 is the initial sample.

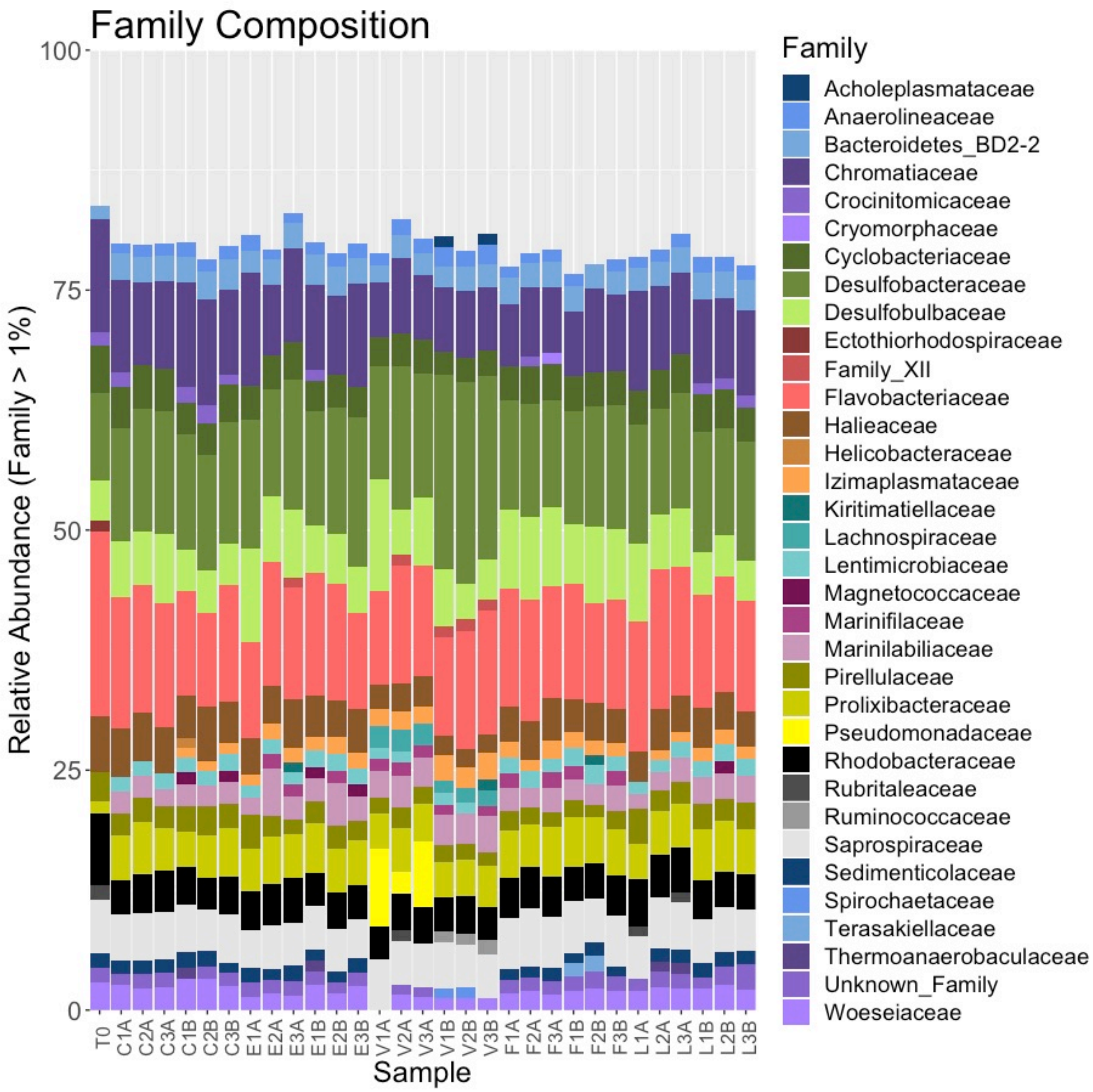

Supplementary Figure 5 Stacked bar plot showing relative abundance for all families present in samples at >1% relative abundance, corresponding to figure 3 in text. Samples are named for polymer type (L = PLA, E = PE, F = PUF, V = PVC and C = Control), replicate (1, 2, or 3) and collection date (A = 7 and B = 16 days), and T0 is the initial sample.

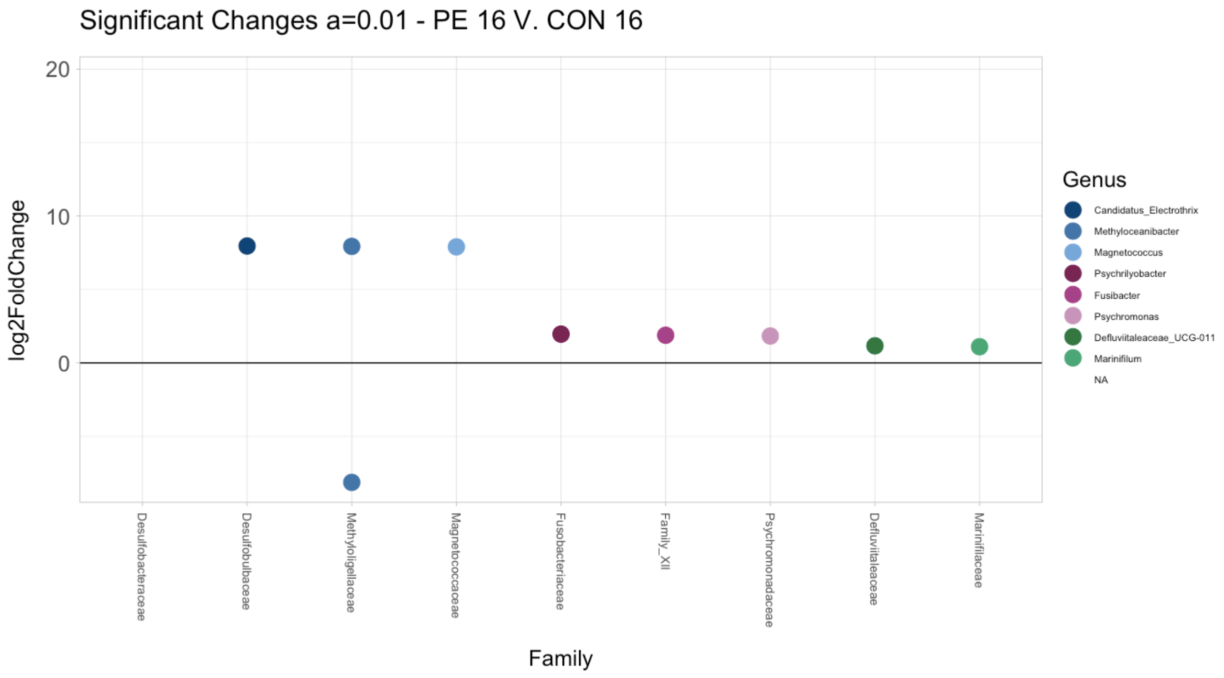

Supplementary Figure 6 DeSeq plot showing the log2 fold change between PE and control treatment communities, organized by family with genus detailed by color. Points above the x-axis are higher in PE treatment, while those below are significantly higher in the control treatment ($\alpha = 0.01$).

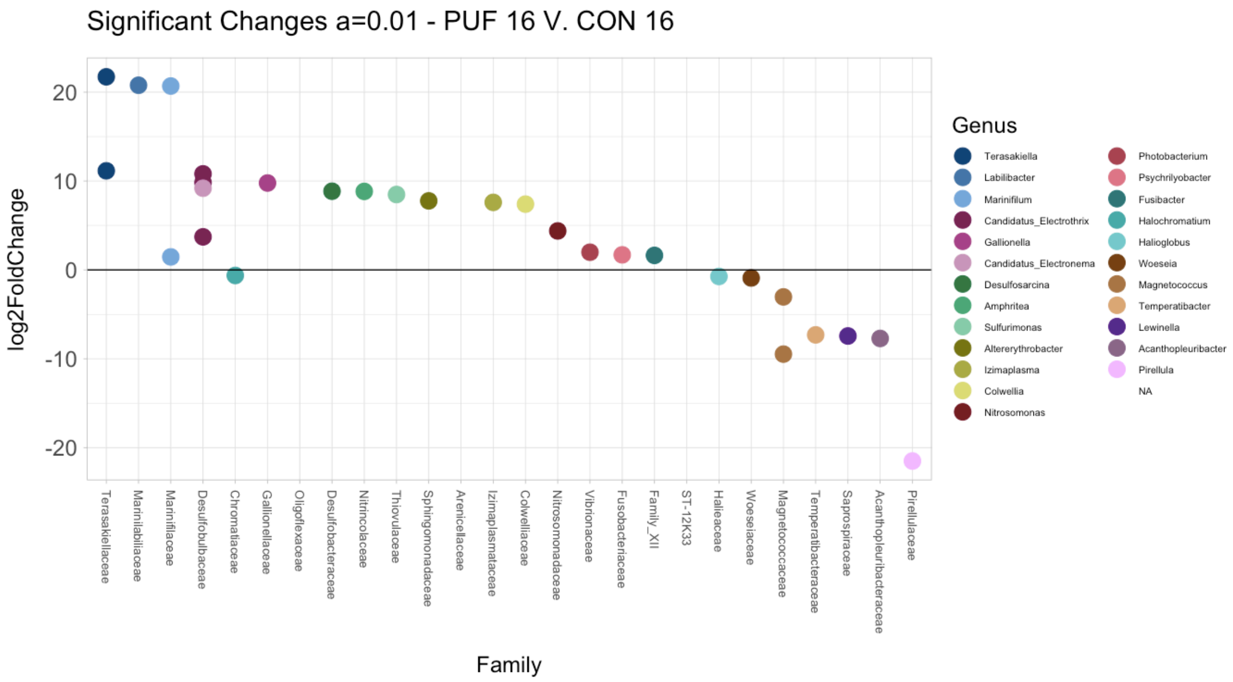

Supplementary Figure 7 DeSeq plot showing the log2 fold change between PUF and control treatment communities, organized by family with genus detailed by color. Points above the x-axis are higher in PUF treatment, while those below are significantly higher in the control treatment (α = 0.01).

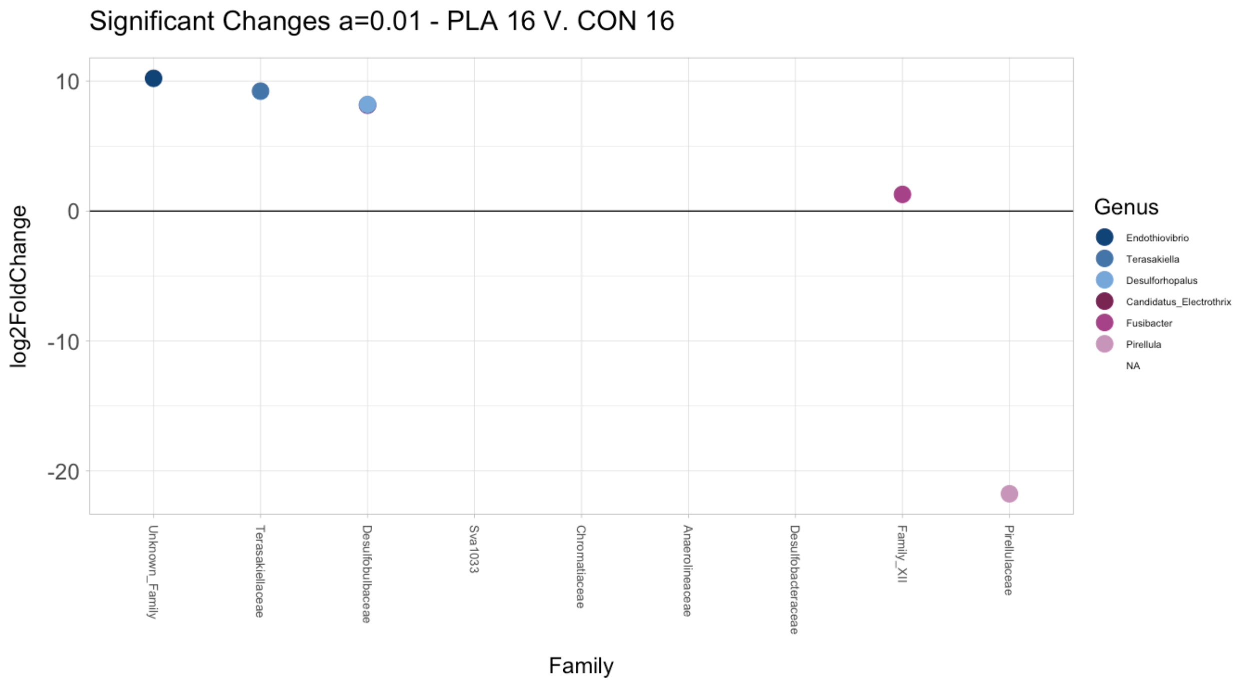

Supplementary Figure 8 DeSeq plot showing the log2 fold change between PLA and control treatment communities, organized by family with genus detailed by color. Points above the x-axis are higher in PLA treatment, while those below are significantly higher in the control treatment (α = 0.01).

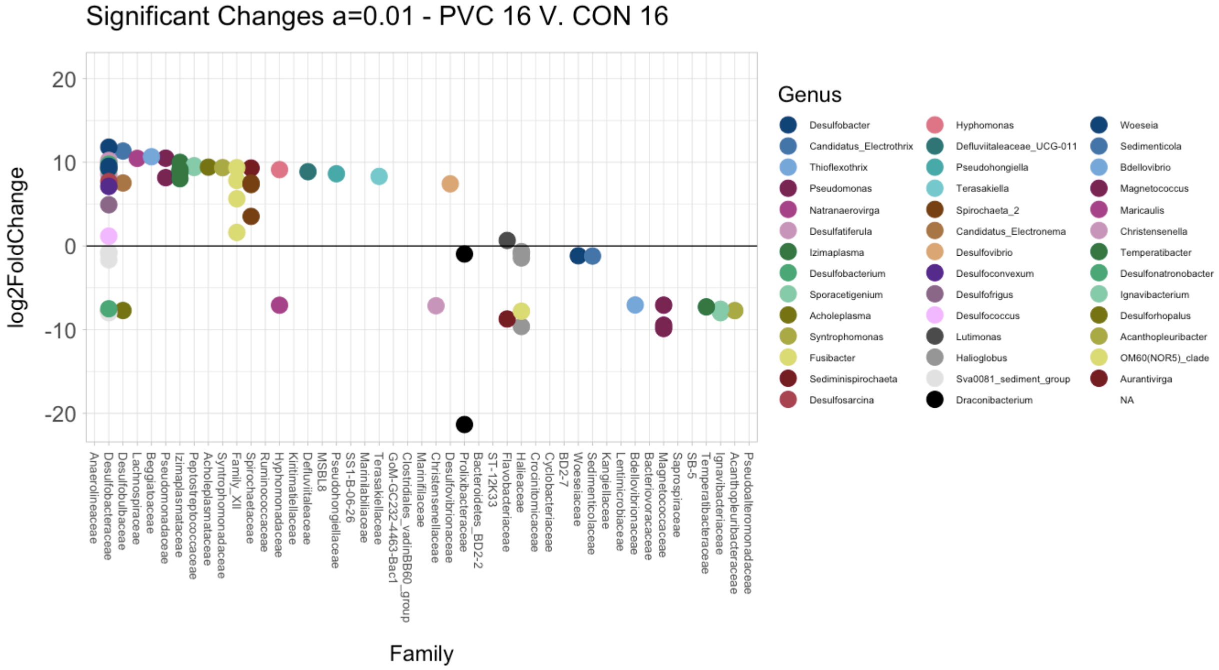

Supplementary Figure 9 DeSeq plot showing the log2 fold change between PVC and control treatment communities, organized by family with genus detailed by color. Points above the x-axis are higher in PVC treatment, while those below are significantly higher in the control treatment (α = 0.01).

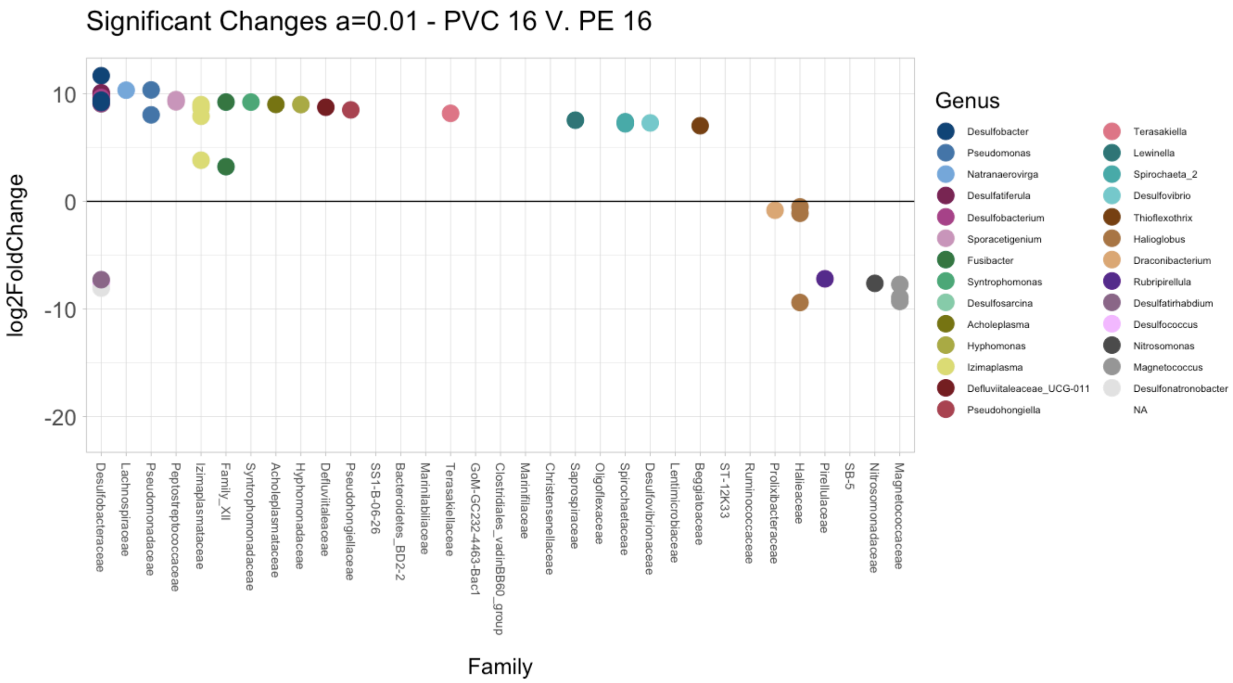

Supplementary Figure 10 DeSeq plot showing the log2 fold change between PVC and PE treatment communities, organized by family with genus detailed by color. Points above the x-axis are higher in PVC treatment, while those below are significantly higher in the PE treatment ($\alpha = 0.01$).

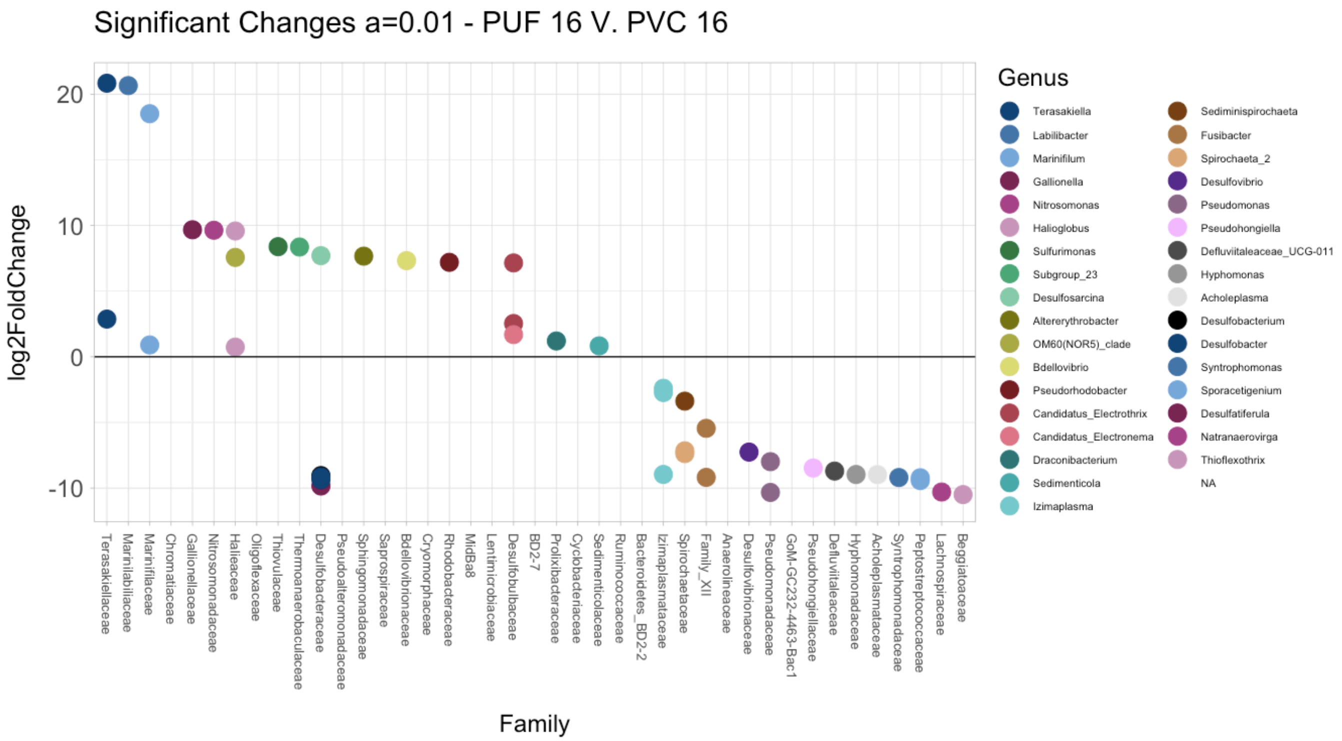

Supplementary Figure 11 DeSeq plot showing the log2 fold change between PUF and PVC treatment communities, organized by family with genus detailed by color. Points above the x-axis are higher in PUF treatment, while those below are significantly higher in the PVC treatment ($\alpha = 0.01$).

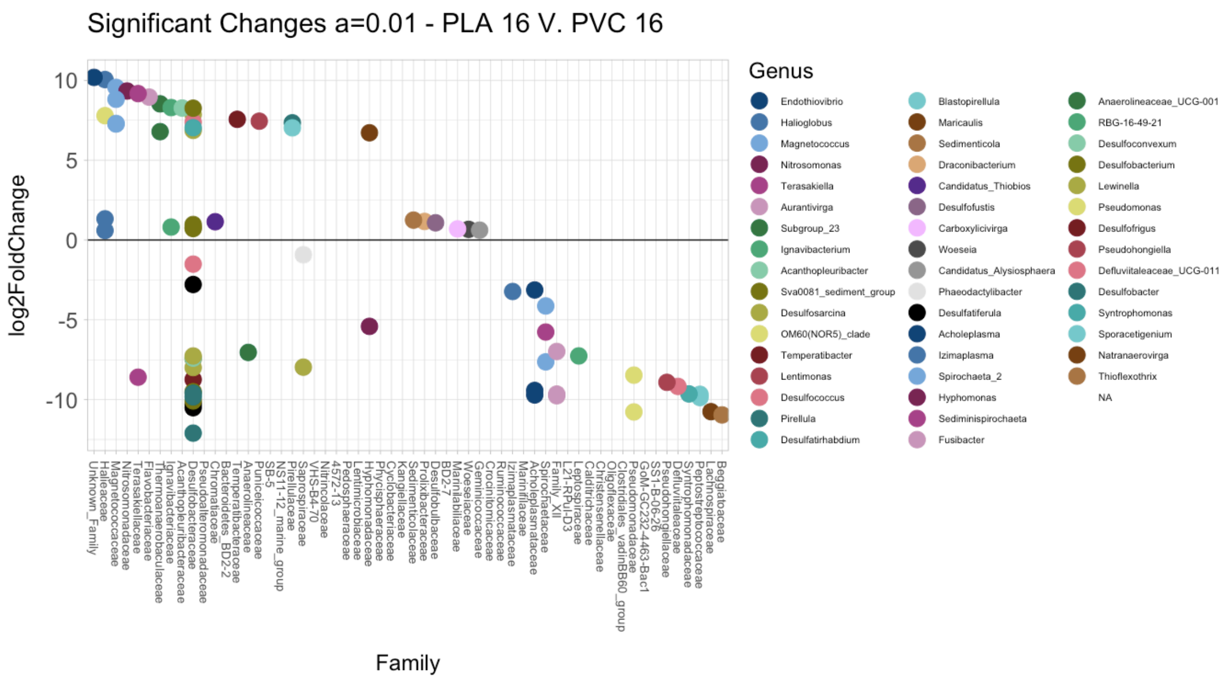

Supplementary Figure 12 DeSeq plot showing the log2 fold change between PLA and PVC treatment communities, organized by family with genus detailed by color. Points above the x-axis are higher in PLA treatment, while those below are significantly higher in the PVC treatment (α = 0.01).

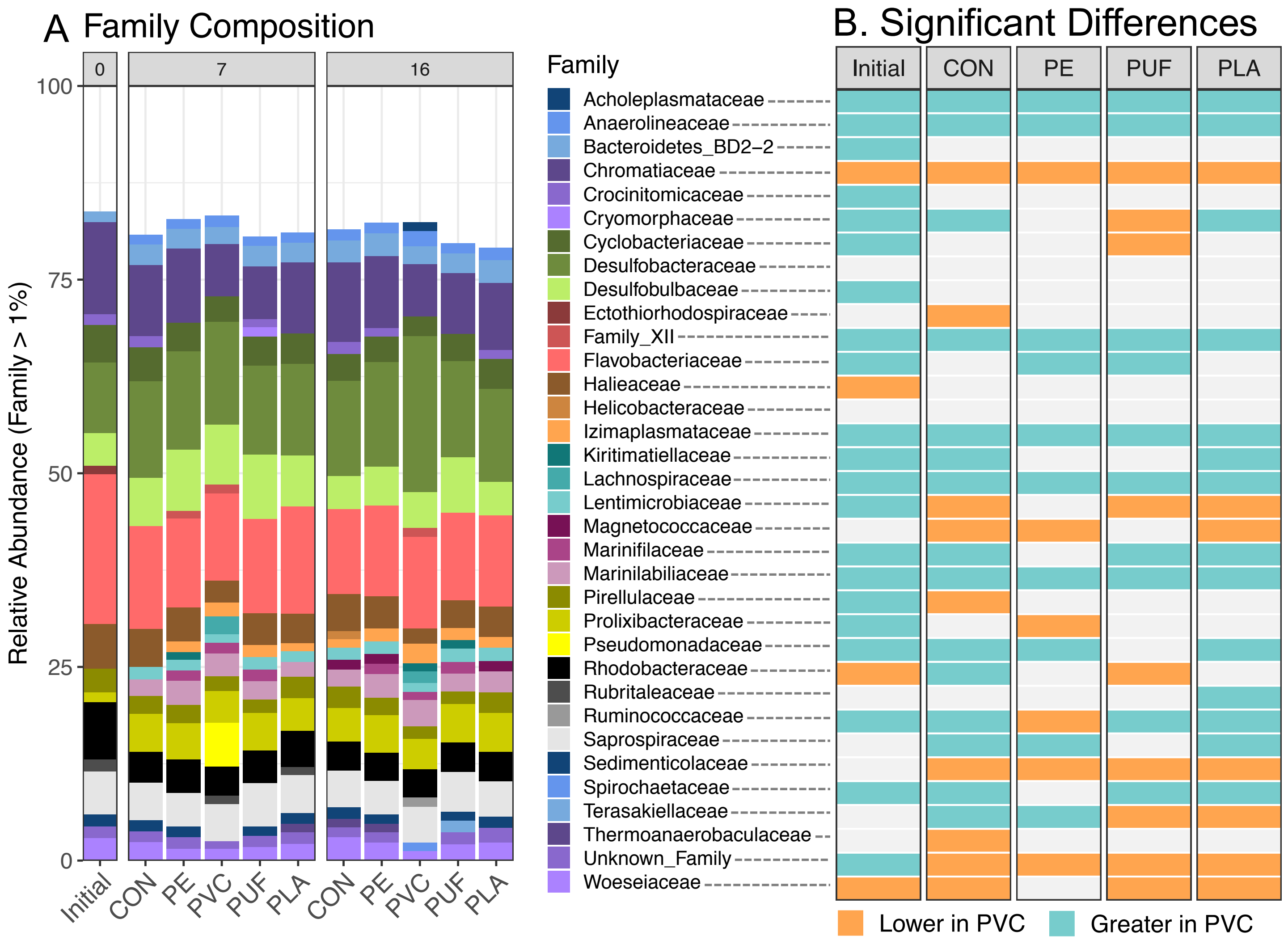

Supplementary Figure 13 A. Stacked bar plot of the relative abundance of families (greater than 1% abundance) for each plastic treatment (averaged for the three replicates) for each sediment collection date (0, 7 and 16 days). B. Families were determined to be significantly between PVC and other treatments (averaged across collection dates) using DeSeq ($\alpha = 0.01$). The left panel shows if each a family is significantly higher in PVC (blue) or the control (orange). In some cases there genera of one family were significantly higher in CON and some were significantly higher in the plastic treatment, in which case no color was assigned.

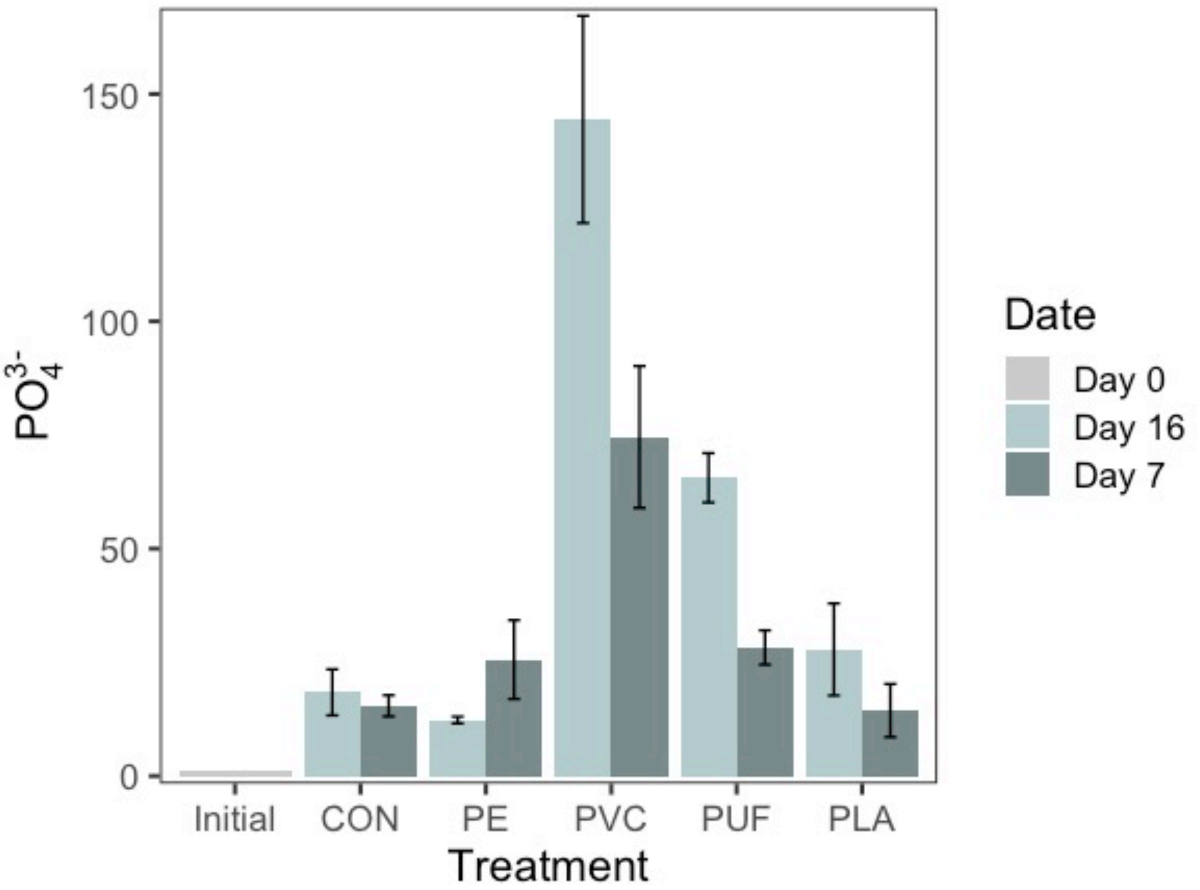

Supplementary Figure 14 Concentration (μM) of phosphate in the water initially and in all treatments after 7 and 16 days incubation (n = 3 per treatment). Error bars are standard error and CON is the control treatment.

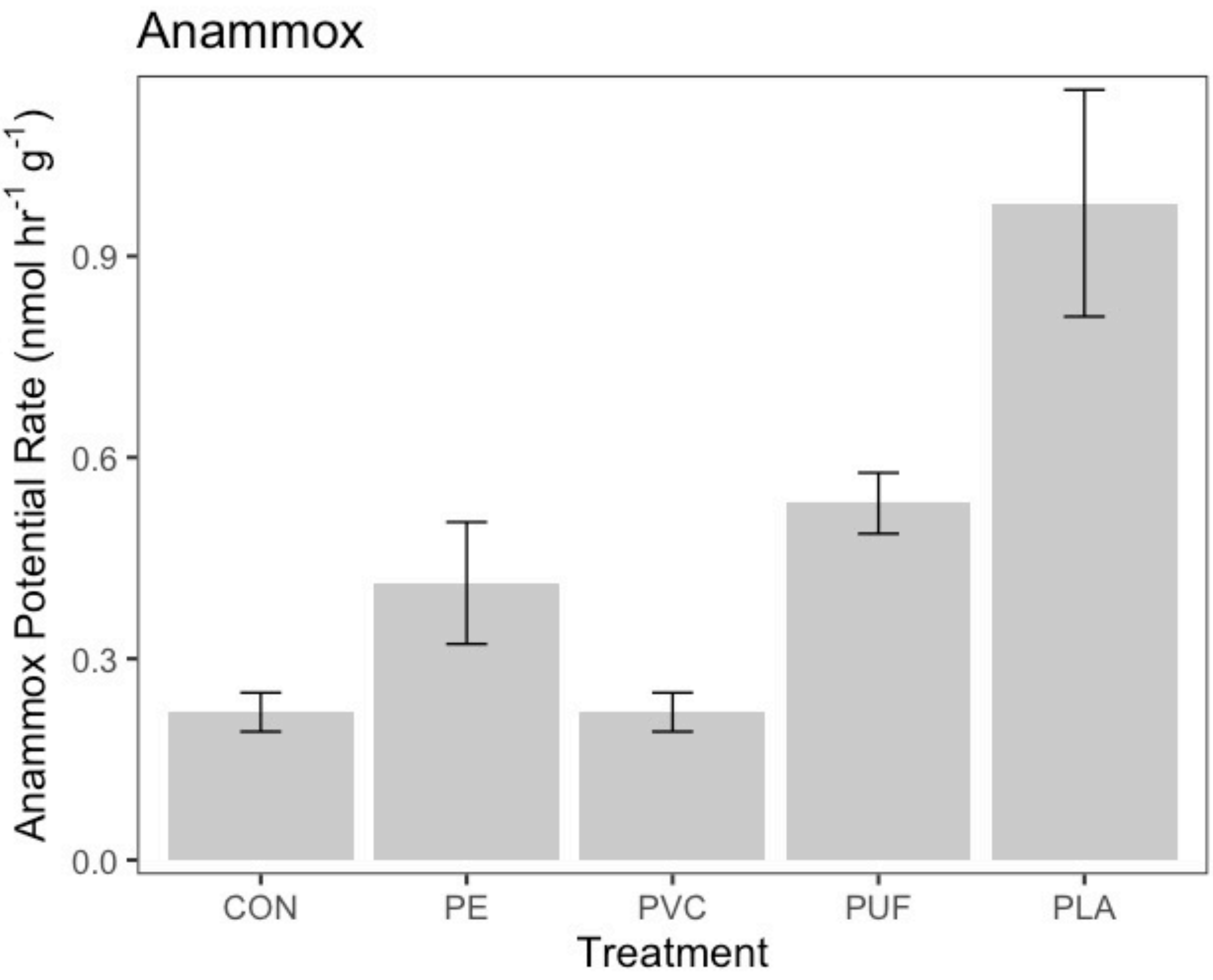

Supplementary Figure 15 Rates of anaerobic ammonium oxidation (Anammox), revealing highest anammox in the biopolymer, PLA and lowest anammox in PVC and the control (n=6 per treatment). Error bars are standard error and CON is the control treatment.

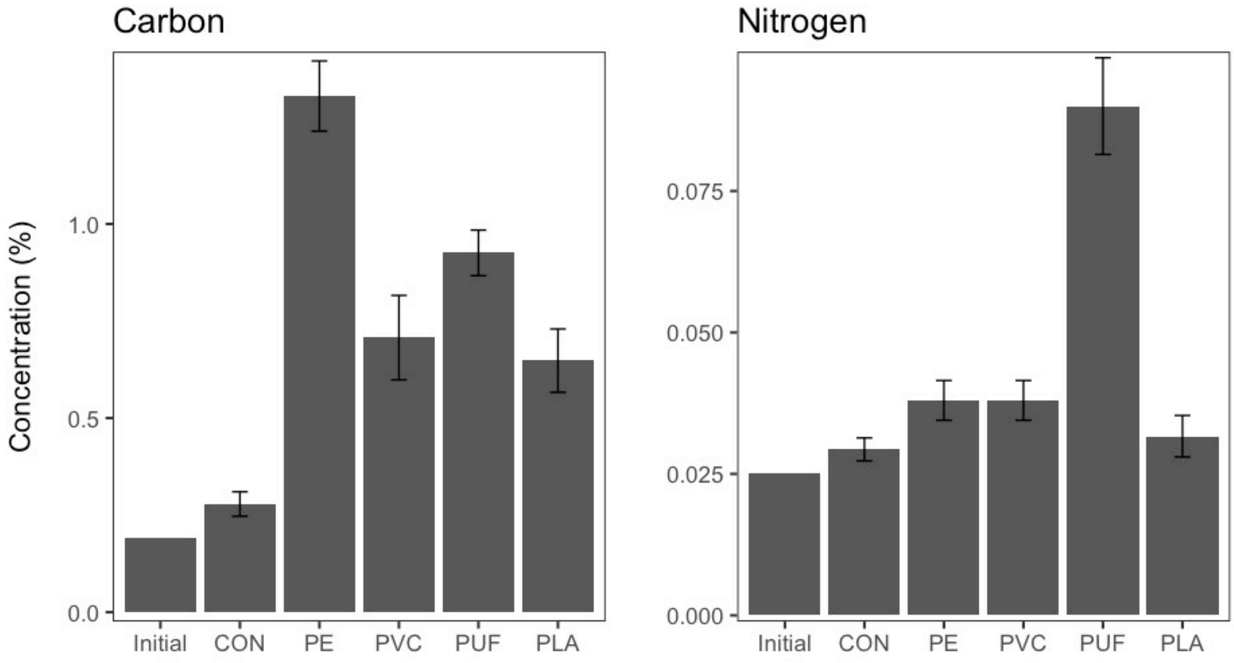

Supplementary Figure 16 The percent organic carbon and nitrogen present in the sediment at the end of the 16 day incubation (n = 3 for each treatment except initial, n =1). Error bars are standard error and CON is the control treatment.

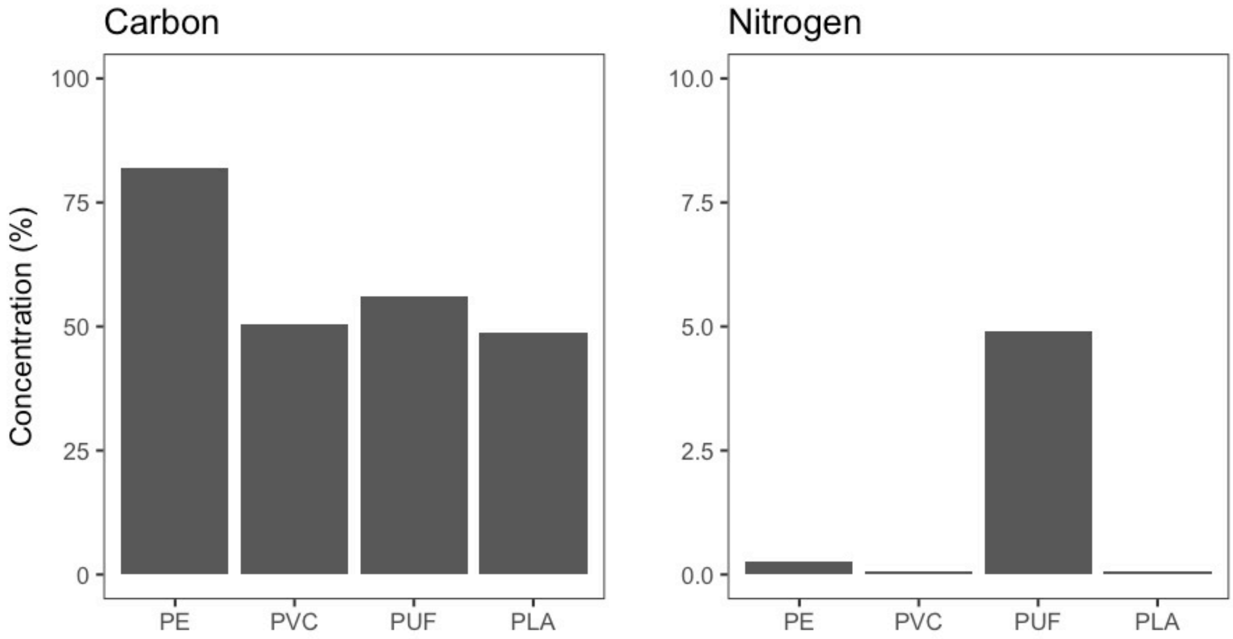

Supplementary Figure 17 The percent organic carbon and nitrogen present in the microplastics.

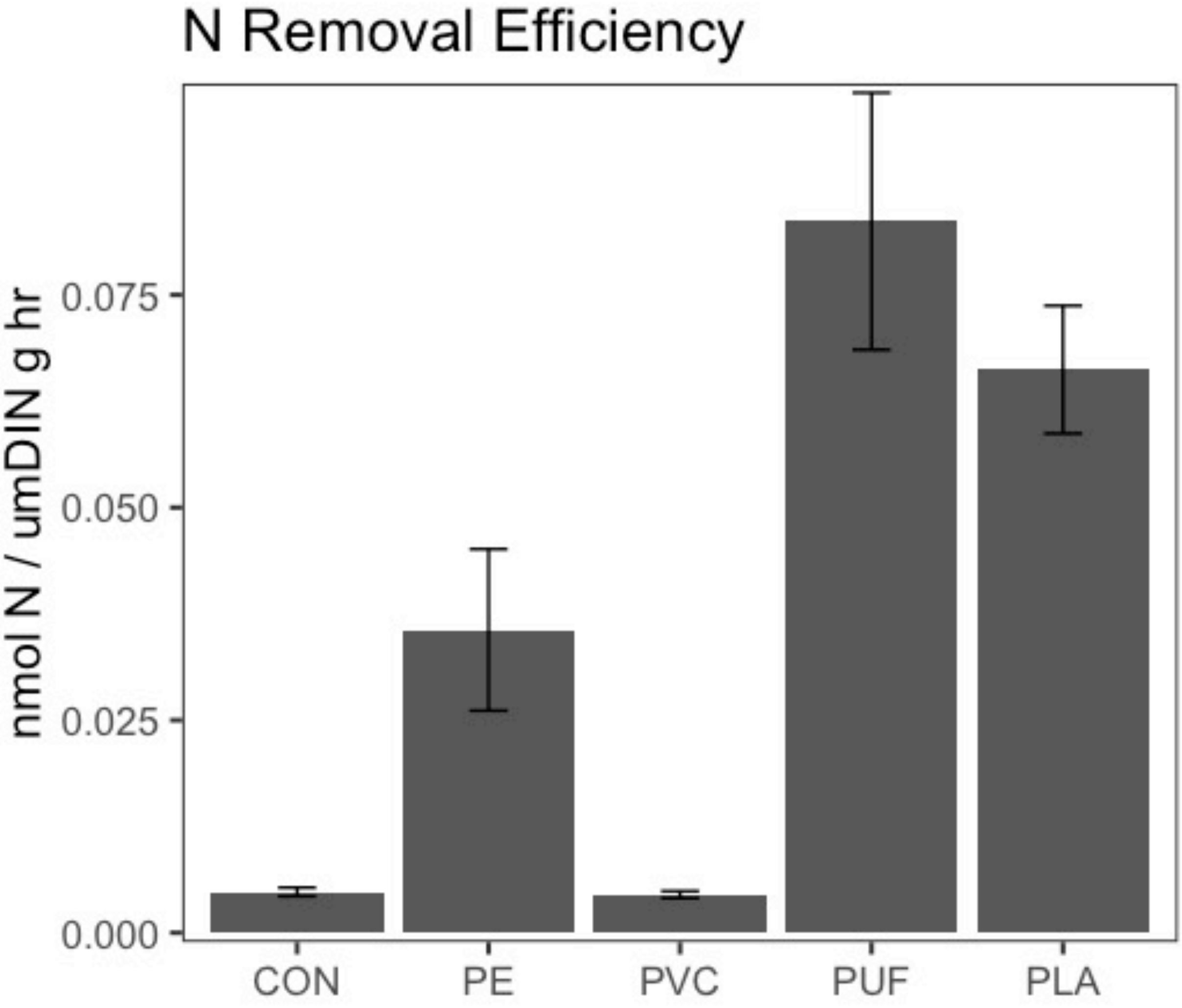

Supplementary Figure 18 The estimated N removal efficiency. Calculated as the sum of anammox and denitrification rates divided by the total DIN in the overlying water, following Semedo and Song (2020). Error bars represent standard error (n = 6 per treatment) and CON is the control treatment.

SUPPLEMENTARY TABLES

Supplementary Table 1 Multivariate permutational ANOVA (PERMANOVA) evaluating community dissimilarity (Fig. 2).

TEST	Parameter	D.F.	Test Statistic	p-value
PERMANOVA	Plastic	5	4.034	0.001
	Day	1	3.323	0.001
	Plastic:Day	4	1.425	0.023
	Residuals	20		
	Total	30		

Supplementary Table 2 NO_3^- in the overlying water column statistical analyses, including normality, homogeneity of variance, two-way ANOVA and post-hoc Tukey (Figure 4).

TEST	Parameter	D.F.	Test Statistic	p-value
Normality (Shaprio -Wilks)			W = 0.68848	1.065E-06
Variance (Levene's)	Treatment	4	F = 2.976	3.869E-02
	Date	1	F = 17.099	2.922E-04
Two-way ANOVA	Treatment	4	F = 3.751	1.960E-02
	Date	1	F = 34.154	1.020E-05
	Treatment:Date	4	F = 3.733	2.000E-02
Post-hoc Tukey Test	PVC:Day 16-CON:Day 7			1.000E+00
	PUF:Day 7-CON:Day 7			1.000E+00
	PE:Day 7-CON:Day 7			1.000E+00
	PLA:Day 7-CON:Day 7			1.000E+00
	PVC:Day 7-CON:Day 7			1.000E+00
	CON:Day 16-CON:Day 7			8.917E-01
	PE:Day 16-CON:Day 7			9.520E-02
	PUF:Day 16-CON:Day 7			6.357E-02
	PLA:Day 16-CON:Day 7			2.025E-03
	PUF:Day 7-PVC:Day 16			1.000E+00
	PE:Day 7-PVC:Day 16			1.000E+00
	PLA:Day 7-PVC:Day 16			1.000E+00
	PVC:Day 7-PVC:Day 16			1.000E+00
	CON:Day 16-PVC:Day 16			8.961E-01
	PE:Day 16-PVC:Day 16			9.742E-02
	PUF:Day 16-PVC:Day 16			6.511E-02
	PLA:Day 16-PVC:Day 16			2.079E-03
	PE:Day 7-PUF:Day 7			1.000E+00
	PLA:Day 7-PUF:Day 7			1.000E+00
	PVC:Day 7-PUF:Day 7			1.000E+00
	CON:Day 16-PUF:Day 7			8.980E-01
	PE:Day 16-PUF:Day 7			9.842E-02
	PUF:Day 16-PUF:Day 7			6.581E-02
	PLA:Day 16-PUF:Day 7			2.103E-03
	PLA:Day 7-PE:Day 7			1.000E+00
	PVC:Day 7-PE:Day 7			1.000E+00
	CON:Day 16-PE:Day 7			8.988E-01
	PE:Day 16-PE:Day 7			9.882E-02
	PUF:Day 16-PE:Day 7			6.609E-02
	PLA:Day 16-PE:Day 7			2.113E-03
	PVC:Day 7-PLA:Day 7			1.000E+00
	CON:Day 16-PLA:Day 7			8.988E-01
	PE:Day 16-PLA:Day 7			9.883E-02
	PUF:Day 16-PLA:Day 7			6.610E-02
	PLA:Day 16-PLA:Day 7			2.113E-03
	CON:Day 16-PVC:Day 7			8.991E-01
	PE:Day 16-PVC:Day 7			9.899E-02
	PUF:Day 16-PVC:Day 7			6.621E-02
	PLA:Day 16-PVC:Day 7			2.117E-03
	PE:Day 16-CON:Day 16			7.550E-01
	PUF:Day 16-CON:Day 16			6.333E-01
	PLA:Day 16-CON:Day 16			4.693E-02
	PUF:Day 16-PE:Day 16			1.000E+00
	PLA:Day 16-PE:Day 16			7.166E-01
	PLA:Day 16-PUF:Day 16			8.273E-01

Supplementary Table 3 NO_2^- in the overlying water column statistical analyses, including normality, homogeneity of variance, two-way ANOVA and post-hoc Tukey (Figure 4).

TEST	Parameter	D.F.	Test Statistic	p-value
Normality (Shaprio -Wilks)			W = 0.70355	1.758E-06
Variance (Levene's)	Treatment	4	F = 10.707	3.394E-05
	Date	1	F = 30.118	7.320E-06
Two-way ANOVA	Treatment	4	F = 30.118	7.290E-06
	Date	1	F = 121.07	6.190E-10
	Treatment:Date	4	F = 15.05	7.900E-06
Post-hoc Tukey Test	PVC:Day 7-CON:Day 7			1
	PUF:Day 7-CON:Day 7			1
	PVC:Day 16-CON:Day 7			1
	PE:Day 7-CON:Day 7			1
	PLA:Day 7-CON:Day 7			1
	CON:Day 16-CON:Day 7			0.82954766
	PE:Day 16-CON:Day 7			3.12E-05
	PUF:Day 16-CON:Day 7			2.74E-05
	PLA:Day 16-CON:Day 7			7.08E-07
	PUF:Day 7-PVC:Day 7			1
	PVC:Day 16-PVC:Day 7			1
	PE:Day 7-PVC:Day 7			1
	PLA:Day 7-PVC:Day 7			1
	CON:Day 16-PVC:Day 7			0.82960955
	PE:Day 16-PVC:Day 7			3.12E-05
	PUF:Day 16-PVC:Day 7			2.74E-05
	PLA:Day 16-PVC:Day 7			7.09E-07
	PVC:Day 16-PUF:Day 7			1
	PE:Day 7-PUF:Day 7			1
	PLA:Day 7-PUF:Day 7			1
	CON:Day 16-PUF:Day 7			0.83045428
	PE:Day 16-PUF:Day 7			3.13E-05
	PUF:Day 16-PUF:Day 7			2.75E-05
	PLA:Day 16-PUF:Day 7			7.11E-07
	PE:Day 7-PVC:Day 16			1
	PLA:Day 7-PVC:Day 16			1
	CON:Day 16-PVC:Day 16			0.83083821
	PE:Day 16-PVC:Day 16			3.14E-05
	PUF:Day 16-PVC:Day 16			2.76E-05
	PLA:Day 16-PVC:Day 16			7.12E-07
	PLA:Day 7-PE:Day 7			1
	CON:Day 16-PE:Day 7			0.83496583
	PE:Day 16-PE:Day 7			3.19E-05
	PUF:Day 16-PE:Day 7			2.81E-05
	PLA:Day 16-PE:Day 7			7.23E-07
	CON:Day 16-PLA:Day 7			0.84556317
	PE:Day 16-PLA:Day 7			3.35E-05
	PUF:Day 16-PLA:Day 7			2.94E-05
	PLA:Day 16-PLA:Day 7			7.54E-07
	PE:Day 16-CON:Day 16			0.00093171
	PUF:Day 16-CON:Day 16			0.00081147
	PLA:Day 16-CON:Day 16			1.47E-05
	PUF:Day 16-PE:Day 16			1
	PLA:Day 16-PE:Day 16			0.61849747
	PLA:Day 16-PUF:Day 16			0.6571722

Supplementary Table 4 NH_4^+ in the overlying water column statistical analyses, including normality, homogeneity of variance, two-way ANOVA and post-hoc Tukey (Figure 4).

TEST	Parameter	D.F.	Test Statistic	p-value
Normality (Shaprio -Wilks)			W = 0.89355	5.862E-03
Variance (Levene's)	Treatment	4	F = 7.0817	5.896E-04
	Date	1	F = 9.0058	5.604E-03
Two-way ANOVA	Treatment	4	F = 24.201	2.010E-07
	Date	1	F = 2.824	1.080E-01
	Treatment:Date	4	F = 45.382	9.270E-10
Post-hoc Tukey Test	PLA:Day 16-PUF:Day 16			1.000E+00
	PE:Day 16-PUF:Day 16			5.185E-01
	PVC:Day 7-PUF:Day 16			4.868E-01
	CON:Day 7-PUF:Day 16			1.267E-02
	PLA:Day 7-PUF:Day 16			9.757E-03
	PUF:Day 7-PUF:Day 16			2.671E-03
	PE:Day 7-PUF:Day 16			8.431E-04
	CON:Day 16-PUF:Day 16			4.356E-08
	PVC:Day 16-PUF:Day 16			7.452E-09
	PE:Day 16-PLA:Day 16			6.784E-01
	PVC:Day 7-PLA:Day 16			6.463E-01
	CON:Day 7-PLA:Day 16			2.221E-02
	PLA:Day 7-PLA:Day 16			1.718E-02
	PUF:Day 7-PLA:Day 16			4.748E-03
	PE:Day 7-PLA:Day 16			1.495E-03
	CON:Day 16-PLA:Day 16			6.657E-08
	PVC:Day 16-PLA:Day 16			1.106E-08
	PVC:Day 7-PE:Day 16			1.000E+00
	CON:Day 7-PE:Day 16			5.796E-01
	PLA:Day 7-PE:Day 16			5.063E-01
	PUF:Day 7-PE:Day 16			2.153E-01
	PE:Day 7-PE:Day 16			8.261E-02
	CON:Day 16-PE:Day 16			1.756E-06
	PVC:Day 16-PE:Day 16			2.299E-07
	CON:Day 7-PVC:Day 7			6.122E-01
	PLA:Day 7-PVC:Day 7			5.384E-01
	PUF:Day 7-PVC:Day 7			2.351E-01
	PE:Day 7-PVC:Day 7			9.155E-02
	CON:Day 16-PVC:Day 7			1.938E-06
	PVC:Day 16-PVC:Day 7			2.519E-07
	PLA:Day 7-CON:Day 7			1.000E+00
	PUF:Day 7-CON:Day 7			9.991E-01
	PE:Day 7-CON:Day 7			9.558E-01
	CON:Day 16-CON:Day 7			9.952E-05
	PVC:Day 16-CON:Day 7			9.821E-06
	PUF:Day 7-PLA:Day 7			9.998E-01
	PE:Day 7-PLA:Day 7			9.764E-01
	CON:Day 16-PLA:Day 7			1.282E-04
	PVC:Day 16-PLA:Day 7			1.245E-05
	PE:Day 7-PUF:Day 7			9.999E-01
	CON:Day 16-PUF:Day 7			4.539E-04
	PVC:Day 16-PUF:Day 7			4.100E-05
	CON:Day 16-PE:Day 7			1.428E-03
	PVC:Day 16-PE:Day 7			1.224E-04
	PVC:Day 16-CON:Day 16			9.733E-01

Supplementary Table 5 PO_4^{2-} in the overlying water statistical analyses, including normality, homogeneity of variance, two-way ANOVA and post-hoc Tukey.

TEST	Parameter	D.F.	Test Statistic	p-value
Normality (Shaprio -Wilks)			W = 0.76875	1.434E-05
Variance (Levene's)	Treatment	5	F = 3.2391	2.168E-02
	Date	2	F = 1.7081	1.996E-01
Two-way ANOVA	Treatment	4	F = 3.2391	6.670E-08
	Date	1	F = 11.588	2.810E-03
	Treatment:Date	4	F = 5.006	5.830E-03
Post-hoc Tukey Test	PE:Day 16-Initial:Day 0			1.000E+00
	PLA:Day 7-Initial:Day 0			1.000E+00
	CON:Day 7-Initial:Day 0			1.000E+00
	CON:Day 16-Initial:Day 0			1.000E+00
	PE:Day 7-Initial:Day 0			9.979E-01
	PLA:Day 16-Initial:Day 0			9.947E-01
	PUF:Day 7-Initial:Day 0			9.938E-01
	PUF:Day 16-Initial:Day 0			2.243E-01
	PVC:Day 7-Initial:Day 0			1.039E-01
	PVC:Day 16-Initial:Day 0			9.138E-05
	PLA:Day 7-PE:Day 16			1.000E+00
	CON:Day 7-PE:Day 16			1.000E+00
	CON:Day 16-PE:Day 16			1.000E+00
	PE:Day 7-PE:Day 16			9.999E-01
	PLA:Day 16-PE:Day 16			9.994E-01
	PUF:Day 7-PE:Day 16			9.992E-01
	PUF:Day 16-PE:Day 16			8.802E-02
	PVC:Day 7-PE:Day 16			2.572E-02
	PVC:Day 16-PE:Day 16			1.685E-06
	CON:Day 7-PLA:Day 7			1.000E+00
	CON:Day 16-PLA:Day 7			1.000E+00
	PE:Day 7-PLA:Day 7			1.000E+00
	PLA:Day 16-PLA:Day 7			9.999E-01
	PUF:Day 7-PLA:Day 7			9.999E-01
	PUF:Day 16-PLA:Day 7			1.151E-01
	PVC:Day 7-PLA:Day 7			3.450E-02
	PVC:Day 16-PLA:Day 7			2.173E-06
	CON:Day 16-CON:Day 7			1.000E+00
	PE:Day 7-CON:Day 7			1.000E+00
	PLA:Day 16-CON:Day 7			1.000E+00
	PUF:Day 7-CON:Day 7			9.999E-01
	PUF:Day 16-CON:Day 7			1.309E-01
	PVC:Day 7-CON:Day 7			3.981E-02
	PVC:Day 16-CON:Day 7			2.464E-06
	PE:Day 7-CON:Day 16			1.000E+00
	PLA:Day 16-CON:Day 16			1.000E+00
	PUF:Day 7-CON:Day 16			1.000E+00
	PUF:Day 16-CON:Day 16			1.878E-01
	PVC:Day 7-CON:Day 16			5.993E-02
	PVC:Day 16-CON:Day 16			3.566E-06
	PLA:Day 16-PE:Day 7			1.000E+00
	PUF:Day 7-PE:Day 7			1.000E+00
	PUF:Day 16-PE:Day 7			4.025E-01
	PVC:Day 7-PE:Day 7			1.519E-01
	PVC:Day 16-PE:Day 7			8.884E-06
	PUF:Day 7-PLA:Day 16			1.000E+00
	PUF:Day 16-PLA:Day 16			4.898E-01
	PVC:Day 7-PLA:Day 16			1.980E-01
	PVC:Day 16-PLA:Day 16			1.185E-05
	PUF:Day 16-PUF:Day 7			5.079E-01
	PVC:Day 7-PUF:Day 7			2.083E-01
	PVC:Day 16-PUF:Day 7			1.255E-05
	PVC:Day 7-PUF:Day 16			1.000E+00
	PVC:Day 16-PUF:Day 16			2.304E-03
	PVC:Day 16-PVC:Day 7			8.568E-03

Supplementary Table 6 *AmoA* gene ratio statistical analyses, including normality, homogeneity of variance, two-way ANOVA and post-hoc Tukey (Figure 5).

TEST	Parameter	D.F.	Test Statistic	p-value
Normality (Shaprio -Wilks)			W = 0.73437	3.895E-06
Variance (Levene's)	Treatment	5	F = 4.51541	6.957E-03
	Date	2	F = 4.7398	1.687E-02
Two-way ANOVA	Treatment	5	F = 3.629	1.690E-02
	Date	1	F = 31.606	1.670E-05
	Treatment:Date	4	F = 6.554	5.140E-03
Post-hoc Tukey Test	CON:Day 16-Initial:Day 0			1
	PE:Day 16-Initial:Day 0			0.99997538
	PLA:Day 16-Initial:Day 0			0.62064767
	PUF:Day 16-Initial:Day 0			0.05606539
	PVC:Day 16-Initial:Day 0			1
	CON:Day 7-Initial:Day 0			1
	PE:Day 7-Initial:Day 0			1
	PLA:Day 7-Initial:Day 0			1
	PUF:Day 7-Initial:Day 0			0.9999994
	PVC:Day 7-Initial:Day 0			1
	PE:Day 16-CON:Day 16			0.999969
	PLA:Day 16-CON:Day 16			0.26392176
	PUF:Day 16-CON:Day 16			0.003866
	PVC:Day 16-CON:Day 16			1
	CON:Day 7-CON:Day 16			0.99999998
	PE:Day 7-CON:Day 16			0.9999994
	PLA:Day 7-CON:Day 16			0.99999933
	PUF:Day 7-CON:Day 16			0.99684812
	PVC:Day 7-CON:Day 16			0.99999675
	PLA:Day 16-PE:Day 16			0.73560763
	PUF:Day 16-PE:Day 16			0.02319693
	PVC:Day 16-PE:Day 16			0.9986454
	CON:Day 7-PE:Day 16			0.99176446
	PE:Day 7-PE:Day 16			0.98084004
	PLA:Day 7-PE:Day 16			0.98031038
	PUF:Day 7-PE:Day 16			0.79183531
	PVC:Day 7-PE:Day 16			0.9701644
	PUF:Day 16-PLA:Day 16			0.76362948
	PVC:Day 16-PLA:Day 16			0.15988391
	CON:Day 7-PLA:Day 16			0.10860366
	PE:Day 7-PLA:Day 16			0.08518213
	PLA:Day 7-PLA:Day 16			0.08443817
	PUF:Day 7-PLA:Day 16			0.02585948
	PVC:Day 7-PLA:Day 16			0.07322598
	PVC:Day 16-PUF:Day 16			0.00204044
	CON:Day 7-PUF:Day 16			0.00129624
	PE:Day 7-PUF:Day 16			0.00098689
	PLA:Day 7-PUF:Day 16			0.00097737
	PUF:Day 7-PUF:Day 16			0.00028324
	PVC:Day 7-PUF:Day 16			0.00083604
	CON:Day 7-PVC:Day 16			1

PE:Day 7-PVC:Day 16	1
PLA:Day 7-PVC:Day 16	1
PUF:Day 7-PVC:Day 16	0.99988027
PVC:Day 7-PVC:Day 16	1
PE:Day 7-CON:Day 7	1
PLA:Day 7-CON:Day 7	1
PUF:Day 7-CON:Day 7	0.99999643
PVC:Day 7-CON:Day 7	1
PLA:Day 7-PE:Day 7	1
PUF:Day 7-PE:Day 7	0.9999998
PVC:Day 7-PE:Day 7	1
PUF:Day 7-PLA:Day 7	0.99999982
PVC:Day 7-PLA:Day 7	1
PVC:Day 7-PUF:Day 7	0.99999998

Supplementary Table 7 *NirS* gene ratio statistical analyses, including normality, homogeneity of variance, two-way ANOVA and post-hoc Tukey (Figure 5).

TEST	Parameter	D.F.	Test Statistic	p-value
Normality (Shaprio -Wilks)			W = 0.97742	7.378E-01
Variance (Levene's)	Treatment	5	F = 0.3826	8.559E-01
	Date	2	F = 0.7885	4.644E-01
Two-way ANOVA	Treatment	5	F = 11.090	3.210E-05
	Date	1	F = 0.00	9.830E-01
	Treatment:Date	4	F = 0.221	9.240E-01
Post-hoc Tukey Test	CON:Day 16-Initial:Day 0			0.15978649
	PE:Day 16-Initial:Day 0			0.13621516
	PLA:Day 16-Initial:Day 0			0.60548759
	PUF:Day 16-Initial:Day 0			0.27450127
	PVC:Day 16-Initial:Day 0			0.00197416
	CON:Day 7-Initial:Day 0			0.19822851
	PE:Day 7-Initial:Day 0			0.17175571
	PLA:Day 7-Initial:Day 0			0.63960867
	PUF:Day 7-Initial:Day 0			0.09923979
	PVC:Day 7-Initial:Day 0			0.00329898
	PE:Day 16-CON:Day 16			1
	PLA:Day 16-CON:Day 16			0.99407658
	PUF:Day 16-CON:Day 16			1
	PVC:Day 16-CON:Day 16			0.25979486
	CON:Day 7-CON:Day 16			1
	PE:Day 7-CON:Day 16			1
	PLA:Day 7-CON:Day 16			0.98953241
	PUF:Day 7-CON:Day 16			1
	PVC:Day 7-CON:Day 16			0.42641165
	PLA:Day 16-PE:Day 16			0.98561727
	PUF:Day 16-PE:Day 16			0.99999984
	PVC:Day 16-PE:Day 16			0.31568153
	CON:Day 7-PE:Day 16			1
	PE:Day 7-PE:Day 16			1
	PLA:Day 7-PE:Day 16			0.97690569
	PUF:Day 7-PE:Day 16			1
	PVC:Day 7-PE:Day 16			0.49983989
	PUF:Day 16-PLA:Day 16			0.99996424
	PVC:Day 16-PLA:Day 16			0.02156645
	CON:Day 7-PLA:Day 16			0.99874386
	PE:Day 7-PLA:Day 16			0.99629147
	PLA:Day 7-PLA:Day 16			1
	PUF:Day 7-PLA:Day 16			0.94685847
	PVC:Day 7-PLA:Day 16			0.04335775
	PVC:Day 16-PUF:Day 16			0.11769376
	CON:Day 7-PUF:Day 16			1
	PE:Day 7-PUF:Day 16			1
	PLA:Day 7-PUF:Day 16			0.99988658
	PUF:Day 7-PUF:Day 16			0.99997677
	PVC:Day 7-PUF:Day 16			0.21411114
	CON:Day 7-PVC:Day 16			0.1943747
	PE:Day 7-PVC:Day 16			0.23655964
	PLA:Day 7-PVC:Day 16			0.01830798
	PUF:Day 7-PVC:Day 16			0.44275699

PVC:Day 7-PVC:Day 16	1
PE:Day 7-CON:Day 7	1
PLA:Day 7-CON:Day 7	0.99736714
PUF:Day 7-CON:Day 7	0.99999995
PVC:Day 7-CON:Day 7	0.33356447
PLA:Day 7-PE:Day 7	0.99310322
PUF:Day 7-PE:Day 7	1
PVC:Day 7-PE:Day 7	0.39431841
PUF:Day 7-PLA:Day 7	0.92580896
PVC:Day 7-PLA:Day 7	0.03698552
PVC:Day 7-PUF:Day 7	0.64828302

Supplementary Table 8 *NirK* gene ratio statistical analyses, including normality, homogeneity of variance, two-way ANOVA and post-hoc Tukey (Figure 5).

TEST	Parameter	D.F.	Test Statistic	p-value
Normality (Shaprio -Wilks)			W = 0.95951	2.832E-01
Variance (Levene's)	Treatment	5	F = 0.6653	6.532E-01
	Date	2	F = 0.5913	5.603E-01
Two-way ANOVA	Treatment	5	F = 1.145	3.700E-01
	Date	1	F = 2.220	1.520E-01
	Treatment:Date	4	F = 0.714	5.920E-01
Post-hoc Tukey Test	CON:Day 16-Initial:Day 0			0.99971571
	PE:Day 16-Initial:Day 0			1
	PLA:Day 16-Initial:Day 0			1
	PUF:Day 16-Initial:Day 0			1
	PVC:Day 16-Initial:Day 0			1
	CON:Day 7-Initial:Day 0			1
	PE:Day 7-Initial:Day 0			1
	PLA:Day 7-Initial:Day 0			1
	PUF:Day 7-Initial:Day 0			0.99997371
	PVC:Day 7-Initial:Day 0			0.99999834
	PE:Day 16-CON:Day 16			0.99920121
	PLA:Day 16-CON:Day 16			0.93859937
	PUF:Day 16-CON:Day 16			0.98786255
	PVC:Day 16-CON:Day 16			0.99997477
	CON:Day 7-CON:Day 16			0.9661984
	PE:Day 7-CON:Day 16			0.80388544
	PLA:Day 7-CON:Day 16			0.94963838
	PUF:Day 7-CON:Day 16			0.48347567
	PVC:Day 7-CON:Day 16			1
	PLA:Day 16-PE:Day 16			0.99999961
	PUF:Day 16-PE:Day 16			1
	PVC:Day 16-PE:Day 16			1
	CON:Day 7-PE:Day 16			0.99999999
	PE:Day 7-PE:Day 16			0.99982564
	PLA:Day 7-PE:Day 16			0.99999987
	PUF:Day 7-PE:Day 16			0.97834607
	PVC:Day 7-PE:Day 16			0.99999933
	PUF:Day 16-PLA:Day 16			1
	PVC:Day 16-PLA:Day 16			0.9999452
	CON:Day 7-PLA:Day 16			1
	PE:Day 7-PLA:Day 16			1
	PLA:Day 7-PLA:Day 16			1
	PUF:Day 7-PLA:Day 16			0.99994282
	PVC:Day 7-PLA:Day 16			0.99590878
	PVC:Day 16-PUF:Day 16			0.99999986
	CON:Day 7-PUF:Day 16			1
	PE:Day 7-PUF:Day 16			0.99999934
	PLA:Day 7-PUF:Day 16			1
	PUF:Day 7-PUF:Day 16			0.99797473
	PVC:Day 7-PUF:Day 16			0.99982422
	CON:Day 7-PVC:Day 16			0.99999342
	PE:Day 7-PVC:Day 16			0.99712222
	PLA:Day 7-PVC:Day 16			0.99997267
	PUF:Day 7-PVC:Day 16			0.92372424

PVC:Day 7-PVC:Day 16	1
PE:Day 7-CON:Day 7	0.99999999
PLA:Day 7-CON:Day 7	1
PUF:Day 7-CON:Day 7	0.99968455
PVC:Day 7-CON:Day 7	0.99870355
PLA:Day 7-PE:Day 7	1
PUF:Day 7-PE:Day 7	0.99999995
PVC:Day 7-PE:Day 7	0.96227042
PUF:Day 7-PLA:Day 7	0.99989127
PVC:Day 7-PLA:Day 7	0.99720391
PVC:Day 7-PUF:Day 7	0.7534891

Supplementary Table 9 Potential denitrification rate statistical analyses, including normality, homogeneity of variance, one-way ANOVA and post-hoc Tukey (Figure 6).

TEST	Parameter	D.F.	Test Statistic	p-value
Normality (Shaprio -Wilks)			W = 0.93972	8.940E-02
Variance (Levene's)	Treatment	4	F = 0.8949	4.817E-01
One-way ANOVA	Treatment	4	F = 16.35	1.060E-06
Post-hoc Tukey Test	PVC-CON			1.000E+00
	PE-CON			9.321E-02
	PUF-CON			2.779E-04
	PLA-CON			1.464E-05
	PE-PVC			9.321E-02
	PUF-PVC			2.779E-04
	PLA-PVC			1.464E-05
	PUF-PE			1.401E-01
	PLA-PE			1.091E-02
	PLA-PUF			7.676E-01

Supplementary Table 10 Potential anammox rate statistical analyses, including normality, homogeneity of variance, one-way ANOVA and post-hoc Tukey.

TEST	Parameter	D.F.	Test Statistic	p-value
Normality (Shaprio -Wilks)			W = 0.81662	1.337E-04
Variance (Levene's)	Treatment	4	F = 1.5575	2.165E-01
One-way ANOVA	Treatment	4	F = 12.04	1.360E-05
Post-hoc Tukey Test	PVC-CON			1.000E+00
	PE-CON			5.653E-01
	PUF-CON			1.364E-01
	PLA-CON			2.968E-05
	PE-PVC			5.653E-01
	PUF-PVC			1.364E-01
	PLA-PVC			2.968E-05
	PUF-PE			8.810E-01
	PLA-PE			1.355E-03
	PLA-PUF			1.351E-02

Supplementary Table 11 Carbon in the sediment statistical analyses, including normality, homogeneity of variance, one-way ANOVA and post-hoc Tukey.

TEST	Parameter	D.F.	Test Statistic	p-value
Normality (Shaprio -Wilks)			W = 0.95916	6.465E-01
Variance (Levene's)	Treatment	5	F = 0.3178	8.912E-01
One-way ANOVA	Treatment	5	F = 22.66	3.710E-05
Post-hoc Tukey Test	CON-Initial			9.927E-01
	PLA-Initial			1.212E-01
	PVC-Initial			6.893E-02
	PUF-Initial			8.622E-03
	PE-Initial			3.024E-04
	PLA-CON			6.384E-02
	PVC-CON			2.843E-02
	PUF-CON			1.729E-03
	PE-CON			2.934E-05
	PVC-PLA			9.934E-01
	PUF-PLA			2.140E-01
	PE-PLA			1.153E-03
	PUF-PVC			4.244E-01
	PE-PVC			2.323E-03
	PE-PUF			3.979E-02

Supplementary Table 12 Nitrogen in the sediment statistical analyses, including normality, homogeneity of variance, one-way ANOVA and post-hoc Tukey.

TEST	Parameter	D.F.	Test Statistic	p-value
Normality (Shaprio -Wilks)			W = 0.72733	3.432E-04
Variance (Levene's)	Treatment	5	F = 0.3611	8.638E-01
One-way ANOVA	Treatment	5	F = 23.22	3.320E-05
Post-hoc Tukey Test	CON-Initial			9.969E-01
	PLA-Initial			9.785E-01
	PE-Initial			7.513E-01
	PVC-Initial			7.513E-01
	PUF-Initial			5.085E-04
	PLA-CON			9.992E-01
	PE-CON			7.909E-01
	PVC-CON			7.909E-01
	PUF-CON			4.728E-05
	PE-PLA			9.288E-01
	PVC-PLA			9.288E-01
	PUF-PLA			6.687E-05
	PVC-PE			1.000E+00
	PUF-PE			1.811E-04
	PUF-PVC			1.811E-04

Supplementary Table 13 All species present in samples at great than 0.1% abundance (separate attachment).

Supplementary Table 14 Genera present in samples at greater than 1% abundance (separate attachment).

Supplementary Table 15 Mean concentrations and standard deviations of flame retardant additives in PUF.

PUF: Concentration (mg/g)		
	mean	std dev
BDE-47	10.9	5.4
BDE-100	3.1	1.0
BDE-99	10.3	2.1
BDE-154	1.5	1.1
BDE-153	1.9	1.1
PentaBDE Total	27.8	
TBB	2.4	0.5
TBPH	0.8	0.3
TCEP	ND	
TCPP	ND	
TDCPP	0.5	0.1
triphenyl phosphate (TPP)	3.1	2.0

SUPPLEMENTARY CODE

```
library(dada2); packageVersion("dada2")
library(magrittr)
library(ggplot2)
library(vegan)
library(plyr)
library(dplyr)
library(scales)
library(grid)
library(reshape2)
library(phyloseq)
library(Rmisc)
library(permute)
library(lattice)

####DADA2 Processing####

fnFs <- sort(list.files(path, pattern="_R1_001.fastq", full.name = TRUE))
fnRs <- sort(list.files(path, pattern="_R2_001.fastq", full.names = TRUE))
sample.names <- sapply(strsplit(basename(fnFs), "_"), `[`, 1)

plotQualityProfile(fnFs[1:33]) # 1:number of samples
plotQualityProfile(fnRs[1:33])

filt_path <- file.path(path, "filtered") # Place filtered files in filtered/ subdirectory
filtFs <- file.path(filt_path, paste0(sample.names, "_F_filt.fastq.gz"))
filtRs <- file.path(filt_path, paste0(sample.names, "_R_filt.fastq.gz"))

out <- filterAndTrim(fnFs, filtFs, fnRs, filtRs, truncLen=c(250,200),
              maxN=0, maxEE=c(2,5), rm.phix=TRUE, trimLeft = c(19,20),
              compress=TRUE, multithread=TRUE) # On Windows set multithread=FALSE
head(out)
errF <- learnErrors(filtFs, multithread=TRUE)
errR <- learnErrors(filtRs, multithread=TRUE)
plotErrors(errF, nominalQ=TRUE)
plotErrors(errR, nominalQ=TRUE)

derepFs <- derepFastq(filtFs, verbose=TRUE)
derepRs <- derepFastq(filtRs, verbose=TRUE)
names(derepFs) <- sample.names
names(derepRs) <- sample.names

dadaFs <- dada(derepFs, err=errF, multithread=TRUE)
dadaRs <- dada(derepRs, err=errR, multithread=TRUE)
dadaFs[[1]]
```

```
mergers <- mergePairs(dadaFs, derepFs, dadaRs, derepRs, verbose=TRUE)
head(mergers[[1]])

seqtab <- makeSequenceTable(mergers)
dim(seqtab)
table(nchar(getSequences(seqtab)))

seqtab.nochim <- removeBimeraDenovo(seqtab, method="consensus", multithread=TRUE,
verbose=TRUE)
dim(seqtab.nochim)
sum(seqtab.nochim)/sum(seqtab)
write.csv(seqtab.nochim, file="seqtab_FR_sed_16S.csv")

getN <- function(x) sum(getUniques(x))
track <- cbind(out, sapply(dadaFs, getN), sapply(mergers, getN), rowSums(seqtab),
rowSums(seqtab.nochim))
colnames(track) <- c("input", "filtered", "denoised", "merged", "tabled", "nonchim")
rownames(track) <- sample.names
head(track)
write.csv(track, file="track_FR_sed_16S.csv")

taxa <- assignTaxonomy(seqtab.nochim,
"/Users/Meredith/Desktop/R/silva_nr_v132_train_set.fa", multithread=TRUE)
taxa <- addSpecies(taxa, "/Users/Meredith/Desktop/R/silva_species_assignment_v132.fa")

taxa.print <- taxa # Removing sequence rownames for display only
rownames(taxa.print) <- NULL
head(taxa.print)
write.csv(taxa, file="FR_sed_16S_taxa.csv")


####Phyloseq####

options(device = "RStudioGD")

library(magrittr)
library(ggplot2)
library(vegan)
library(plyr)
library(dplyr)
library(scales)
library(grid)
library(reshape2)
library(phyloseq)
library(Rmisc)
```

```
library(permute)
library(lattice)

scale_reads <- function(physeq, n) {
  physeq.scale <-
    transform_sample_counts(physeq, function(x) {
      (n * x/sum(x))
    })
  otu_table(physeq.scale) <- floor(otu_table(physeq.scale))
  physeq.scale <- prune_taxa(taxa_sums(physeq.scale) > 0, physeq.scale)
  return(physeq.scale)
}

physeq <- phyloseq(otu_table(seqtab.nochim, taxa_are_rows=FALSE),
                   tax_table(taxa))
physeq

map <- read.csv("/Users/Meredith/Desktop/R/Dada2/AugFR/sed_map_dadaFR_DE.csv",
header=TRUE)
map <- sample_data(map)
rownames(map) <- map$SampleID
head(map)
moth_merge <- merge_phyloseq(physeq, map)

theme_set(theme_bw(base_size = 16))

# removing eukaryotes, archaea, chloroplasts, and mitochondria
erie <- moth_merge %>%
  subset_taxa(
    Kingdom == "Bacteria" &
      Family  != "mitochondria" &
      Class   != "Chloroplast"
  )
erie


a<-sample_sums(moth_merge)
b<-sample_sums(erie)
write.csv(a, "sum.samples.moth_merge.csv")
write.csv(b, "sum.samples.erie1.csv")


# filtering out low abundance ASVs
erie = filter_taxa(erie, function(x) sum(x > 10) > (0.1*length(x)), prune=TRUE)
erie
```

```
b<-sample_sums(erie)
write.csv(b, "sum.samples.erie2.csv")


#Rarefy
min_lib <- min(sample_sums(erie))
set.seed(2)
erie.rare <- rarefy_even_depth(erie, sample.size = min_lib, verbose = FALSE, replace = TRUE)
erie.rare


# Converting your map variables into ordered factors
sample_data(erie)$Treatment <- factor(
  sample_data(erie)$Treatment,
  levels = c("Initial", "Control", "Polyethylene", "Polyvinyl chloride", "Polyurethane foam",
"Polylactic acid")
)

sample_data(erie)$Plastic <- factor(
  sample_data(erie)$Plastic,
  levels = c("Initial", "CON", "PE", "PVC", "PUF", "PLA")
)

sample_data(erie)$Replicate <- factor(
  sample_data(erie)$Replicate,
  levels = c("0","1", "2", "3")
)

sample_data(erie)$Day <- factor(
  sample_data(erie)$Day,
  levels = c("0", "7", "16")
)

sample_data(erie)$SampleID <- factor(sample_data(erie)$SampleID,
                                levels = c("T0", "C1A", "C2A", "C3A", "C1B", "C2B", "C3B", "E1A",
"E2A", "E3A", "E1B", "E2B", "E3B", "V1A", "V2A", "V3A", "V1B", "V2B", "V3B", "F1A",
"F2A", "F3A", "F1B", "F2B", "F3B", "L1A", "L2A", "L3A",  "L1B", "L2B", "L3B"))


sample_data(erie)$Condition <- factor(
  sample_data(erie)$Condition,
  levels = c("Initial", "CON 7", "CON 16", "PE 7", "PE 16", "PVC 7", "PVC 16", "PUF 7", "PUF
16", "PLA 7", "PLA 16")
)
```

```
#### Figure 2 ####
erie_PCoA_rare <- ordinate(
  physeq = erie.rare,
  method = "PCoA",
  distance = "bray")

theme_set(theme_bw(base_size = 16))

p <- plot_ordination(
  physeq = erie,
  ordination = erie_PCoA_rare,
  color = "Plastic",
  shape = "Day",
  title = "Principal Coordinate Analysis") +
  scale_color_manual(values = c("slategray4", "indianred2", "darkolivegreen3",
"mediumpurple3", "tan1", "cornflowerblue"))+
  scale_shape_manual(values=c(16, 16, 16)) +
  geom_point(aes(color = Plastic), size = 6, alpha = 0.75) +           #### Outside symbol
  theme(legend.title = element_text(size = 20))+
  theme(legend.position="right")+
  theme(legend.text = element_text(size = 18)) +
  theme(plot.title=element_text(size=24, margin=margin(0,0,20,0))) +
  theme(axis.title.x = element_text(size=20, margin=margin(15,0,0,0)),
        axis.text.x  = element_text(size=16, margin=margin(5,0,0,0))) +
  theme(axis.title.y = element_text(size=20, margin=margin(0,15,0,0)),
        axis.text.y  = element_text(size=16, margin=margin(0,5,0,0))) +
  theme(axis.title.x = element_text(vjust=-0.5)) +
  theme(axis.title.y = element_text(vjust=1.0)) +
  theme(plot.margin = unit(c(1,1,1,1), "cm"))
p

#Permanova
# Calculate bray curtis distance matrix
erie_rare_bray <- phyloseq::distance(erie.rare, method = "bray")

# make a data frame from the sample_data
sampledf <- data.frame(sample_data(erie.rare))

# Adonis test (PERMANOVA itself)
adonis(erie_rare_bray ~ Plastic*Day, data = sampledf)



#### Figure 3 #####
#Family >1%
erie_Family1 <- erie %>%
```

```
  tax_glom(taxrank = "Family") %>%
  transform_sample_counts(function(x) {(x/sum(x))*100} ) %>%
  psmelt() %>%
  filter(Abundance > 1) %>%
  arrange(Family)

Family1_Factor <- summarySE(erie_Family1, measurevar="Abundance",
                            groupvars=c("Family", "Plastic", "Day"),
                            na.rm=TRUE)
Family1_Factor

ggplot(Family1_Factor, aes(x = Plastic, y = Abundance, fill = Family)) +
  geom_bar(stat = "identity") +
  scale_fill_manual(values = family_colors) +
  theme(axis.title.x = element_blank(),
        axis.title.y = element_text(size=18),
        axis.text.x = element_text(size=18, angle=45, hjust = 1),
        axis.text.y = element_text(size=16),
        legend.text = element_text(size=14),
        plot.title = element_text(size=24))+
  ylab("Relative Abundance (Family > 1%)") +
  xlab("Treatment")+
  theme(legend.text = element_text(size = 14), legend.title = element_text(size = 18))+
  theme(legend.key.height = unit(0.6,"cm"))+
  facet_grid(~Day, scales = "free", space = "free_x")+
  guides(fill = guide_legend(ncol=1, reverse = FALSE))+
  ggtitle("Family Composition")+
  scale_y_continuous(limits = c(0,100))+
  coord_cartesian(ylim = c(0,100), expand=FALSE)+
  theme(plot.margin = unit(c(0.5,0.5,0.5,0.5), "cm"))

##Heat Map
datagg2 <- read.csv("Heatmap_GG2.csv")
View(datagg2)
ggplot(data = datagg2, mapping = aes(x = Treatment,
                                     y = Families,
                                     fill = Significant.Differences)) +
  geom_tile(color = "white", size = 0.5) +
  scale_y_discrete(limits = rev(levels(datagg2$Families)))+
  theme(axis.title.y = element_text(size=18),
        axis.text.x = element_blank(),
        axis.title.x = element_blank(),
        axis.ticks.x = element_blank(),
        axis.text.y = element_text(size=16),
        legend.text = element_text(size=14),
        legend.title = element_text(size=16),
```

```
      plot.title = element_text(size=24),
      panel.spacing = unit(0.2, "lines"),
      legend.justification = "top")+
  coord_cartesian(expand = FALSE)+
  scale_fill_manual(values = c("indianred3", "#114477", "gray95"))+
  guides(fill = guide_legend(reverse = FALSE, keywidth = 1, keyheight = 1))+
  facet_grid(~Treatment, scales = "free", space = "free_x")+
  guides(fill = guide_legend(ncol=1, reverse = TRUE))

#Differences between PVC
datagg2PVC <- read.csv("Heatmap_GG2_PVC.csv", header = TRUE)
View(datagg2PVC)
datagg2PVC$Treatment <- factor(datagg2PVC$Treatment, levels = c("Initial", "CON", "PE",
"PUF", "PLA"))
ggplot(data = datagg2PVC, mapping = aes(x = Treatment,
                              y = Families,
                              fill = Significant_Differences)) +
  geom_tile(color = "white", size = 0.5) +
  scale_y_discrete(limits = rev(levels(datagg2$Families)))+
  theme(axis.title.y = element_text(size=18),
      axis.text.x = element_blank(),
      axis.title.x = element_blank(),
      axis.ticks.x = element_blank(),
      axis.text.y = element_text(size=16),
      legend.text = element_text(size=14),
      legend.title = element_text(size=16),
      plot.title = element_text(size=24),
      panel.spacing = unit(0.2, "lines"),
      legend.justification = "top")+
  coord_cartesian(expand = FALSE)+
  scale_fill_manual(values = c("#77CCCC", "tan1", "gray95"))+
  guides(fill = guide_legend(reverse = FALSE, keywidth = 1, keyheight = 1))+
  facet_grid(~Treatment, scales = "free", space = "free_x")+
  guides(fill = guide_legend(ncol=1, reverse = TRUE))


#### Figure 4 ####

setwd("~/Desktop/R/SedMP/NP")

my_NH4F <- read.csv("NH4F.csv")
View (my_NH4F)
my_NH4F$Date <- factor(
  my_NH4F$Date,
  levels = c("Day 0", "Day 7", "Day 16"))
my_NH4Fs <- summarySE(my_NH4F, measurevar="NH4", groupvars=c("Date","Treatment"))
```

```
my_NH4Fs <- my_NH4Fs[-1,]
p2 <- ggplot(data=my_NH4Fs, aes(x = Treatment, y = NH4, fill = Date))+
  geom_bar(stat = "identity", position = position_dodge())+
  theme_bw(base_size = 16)+
  scale_fill_manual(values = c("lightcyan3", "lightcyan4"))+
  theme(panel.grid.minor = element_blank(), panel.grid.major = element_blank())+
  scale_x_discrete(limits=c("CON", "PE", "PVC", "PUF", "PLA"))+
  geom_errorbar(aes(ymin=NH4-se, ymax=NH4+se), width=.2, position = position_dodge(0.9))+
  scale_y_continuous(expand = c(0.01, 0.01))+
  labs(title=expression('NH'[4]^{'+'}))+
  theme(axis.title.y=element_blank(), axis.title.x=element_blank(), legend.position = "none")
p2


my_NO2F <- read.csv("NO2F.csv")
View(my_NO2F)
my_NO2F$Date <- factor(
  my_NO2F$Date,
  levels = c("Day 7", "Day 16"))
my_NO2Fs <- summarySE(my_NO2F, measurevar="NO2", groupvars=c("Date","Treatment"))
my_NO2Fs <- my_NO2Fs[-11,]
p3 <- ggplot(data=my_NO2Fs, aes(x = Treatment, y = NO2, fill = Date))+
  geom_bar(stat = "identity", position = position_dodge())+
  theme_bw(base_size = 16)+
  scale_fill_manual(values = c("lightcyan3", "lightcyan4"))+
  theme(panel.grid.minor = element_blank(),panel.grid.major = element_blank())+
  scale_x_discrete(limits=c("CON", "PE", "PVC", "PUF", "PLA"))+
  geom_errorbar(aes(ymin=NO2-se, ymax=NO2+se), width=.2, position = position_dodge(0.9))+
  scale_y_continuous(expand = c(0.01, 0.01))+
  labs(title=expression('NO'[2]^{'-'}))+xlab("Treatment")+
  theme(axis.title.y=element_blank(), legend.position = "none")
p3


my_NO3F <- read.csv("NO3F.csv")
View(my_NO3F)
my_NO3F$Date <- factor(
  my_NO3F$Date,
  levels = c("Day 0", "Day 7", "Day 16"))
my_NO3Fs <- summarySE(my_NO3F, measurevar="NO3", groupvars=c("Date","Treatment"))
my_NO3Fs <- my_NO3Fs[-1,]
p4 <- ggplot(data=my_NO3Fs, aes(x = Treatment, y = NO3, fill = Date))+
  geom_bar(stat = "identity", position = position_dodge())+
  theme_bw(base_size = 16)+
  scale_fill_manual(values = c("lightcyan3", "lightcyan4"))+
  theme(panel.grid.minor = element_blank(),panel.grid.major = element_blank())+
```

```
  scale_x_discrete(limits=c("CON", "PE", "PVC", "PUF", "PLA"))+
  geom_errorbar(aes(ymin=NO3-se, ymax=NO3+se), width=.2, position = position_dodge(0.9))+
  scale_y_continuous(expand = c(0.01, 0.01))+
  labs(title=expression('NO'[3]^{'-'}))+
  ylab(expression(paste("Concentration (",mu,"M)")))+
  theme( axis.title.x=element_blank(), legend.position = c(0.2, 0.85))
p4

plot_grid(p4, p3, p2, nrow = 1, align = 'hv')

shapiro.test(my_NO3F$NO3)
leveneTest(NO3 ~ Treatment, data=my_NO3F)
leveneTest(NO3 ~ Date, data=my_NO3F)
mod3 = aov(NO3 ~ Treatment*Date, data = my_NO3F)
summary(mod)
res = TukeyHSD(mod, "Treatment:Date", ordered=TRUE)
TukNitrate <- as.data.frame(res$"Treatment:Date")
write.csv(TukNitrate, "TukNitrate.csv")



#### Figure 5 ####

norm16SUPDATED <- read.csv("16ratio_updated.csv")
View(norm16SUPDATED)
percent$Date <- factor(
  percent$Date,
  levels = c("Day 7", "Day 16"))
norm16Ss <- summarySE(norm16SUPDATED, measurevar="amoA",
groupvars=c("Date","Treatment"))
norm16Ss <- norm16Ss[-1,]
p1 <- ggplot(data=norm16Ss, aes(x = Treatment, y = amoA, fill = Date, order = rev(Date)))+
  geom_bar(stat = "identity", position = position_dodge2(reverse = TRUE))+
  theme_bw(base_size = 16)+
  scale_fill_manual(breaks = c("Day 7", "Day 16"),
                 values = c("lightcyan4", "lightcyan3"))+
  theme(panel.grid.minor = element_blank(),panel.grid.major = element_blank())+
  scale_x_discrete(limits=c("CON", "PE", "PVC", "PUF", "PLA"))+
  geom_errorbar(aes(ymin=amoA-se, ymax=amoA+se),
             position = position_dodge2(width = 0.9, padding = 0.6, reverse=TRUE))+
  labs(title="amoA") + theme(plot.title = element_text(face="italic"))+
  scale_y_continuous(expand = c(0.000001, 0.000001))+
  ylab("Gene ratio to 16S")+
  theme(axis.title.x=element_blank(), legend.position = c(0.2, 0.85))
p1
shapiro.test(norm16SUPDATED$amoA)
```

```
leveneTest(amoA ~ Treatment, data=norm16SUPDATED)
leveneTest(amoA ~ Date, data=norm16SUPDATED)
res.aov <- aov(amoA ~ Treatment*Date, data = norm16SUPDATED)
summary(res.aov)
res = TukeyHSD(res.aov)
TukamoA <- as.data.frame(res$"Treatment:Date")
write.csv(TukamoA, "TukamoA.csv")


norm16Ss <- summarySE(norm16SUPDATED, measurevar="nirS",
groupvars=c("Date","Treatment"))
norm16Ss <- norm16Ss[-1,]
p2 <- ggplot(data=norm16Ss, aes(x = Treatment, y = nirS, fill = Date, order = rev(Date)))+
  geom_bar(stat = "identity", position = position_dodge2(reverse = TRUE))+
  theme_bw(base_size = 16)+
  scale_fill_manual(breaks = c("Day 7", "Day 16"),
                    values = c("lightcyan4", "lightcyan3"))+
  theme(panel.grid.minor = element_blank(), panel.grid.major = element_blank())+
  scale_x_discrete(limits=c("CON", "PE", "PVC", "PUF", "PLA"))+
  geom_errorbar(aes(ymin=nirS-se, ymax=nirS+se),
                position = position_dodge2(width = 0.9, padding = 0.6, reverse=TRUE))+
  labs(title="nirS") + theme(plot.title = element_text(face="italic"))+
  scale_y_continuous(expand = c(0.0005, 0.0005))+
  xlab("Treatment")+
  theme(axis.title.y=element_blank(), legend.position = "none")
p2
shapiro.test(norm16SUPDATED$nirS)
leveneTest(nirS ~ Treatment, data=norm16SUPDATED)
leveneTest(nirS ~ Date, data=norm16SUPDATED)
res.aov <- aov(nirS ~ Treatment*Date, data = norm16SUPDATED)
summary(res.aov)
res = TukeyHSD(res.aov)
TuknirS <- as.data.frame(res$"Treatment:Date")
write.csv(TuknirS, "TuknirS.csv")

norm16Ss <- summarySE(norm16SUPDATED, measurevar="nirK",
groupvars=c("Date","Treatment"))
norm16Ss <- norm16Ss[-1,]
p3 <- ggplot(data=norm16Ss, aes(x = Treatment, y = nirK, fill = Date, order = rev(Date)))+
  geom_bar(stat = "identity", position = position_dodge2(reverse = TRUE))+
  theme_bw(base_size = 16)+
  scale_fill_manual(breaks = c("Day 7", "Day 16"),
                    values = c("lightcyan4", "lightcyan3"))+
  theme(panel.grid.minor = element_blank(), panel.grid.major = element_blank())+
  scale_x_discrete(limits=c("CON", "PE", "PVC", "PUF", "PLA"))+
  geom_errorbar(aes(ymin=nirK-se, ymax=nirK+se),
```

```
            position = position_dodge2(width = 0.9, padding = 0.6, reverse=TRUE))+
  labs(title="nirK") + theme(plot.title = element_text(face="italic"))+
  scale_y_continuous(expand = c(0.00001, 0.00001))+
  theme(axis.title.y=element_blank(), axis.title.x=element_blank(), legend.position = "none")
p3
shapiro.test(norm16SUPDATED$nirK)
leveneTest(nirK ~ Treatment, data=norm16SUPDATED)
leveneTest(nirK ~ Date, data=norm16SUPDATED)
res.aov <- aov(nirK ~ Treatment*Date, data = norm16SUPDATED)
summary(res.aov)
res = TukeyHSD(res.aov)
TuknirK <- as.data.frame(res$"Treatment:Date")
write.csv(TuknirK, "TuknirK.csv")

plot_grid(p1, p2, p3, nrow = 1, align = 'hv')



#### Figure 6 ####

my_ratess <- summarySE(my_rates, measurevar="Denitrification", groupvars=c("Treatment"))
ggplot(data=my_ratess, aes(x = Treatment, y = Denitrification))+
  geom_bar(stat = "identity", position = position_dodge(), fill="gray80")+
  theme_bw(base_size = 16)+
  theme(panel.grid.minor = element_blank(),panel.grid.major = element_blank())+
  scale_x_discrete(limits=c("CON", "PE", "PVC", "PUF", "PLA"))+
  geom_errorbar(aes(ymin=Denitrification-se, ymax=Denitrification+se), width=.2, position = 
position_dodge(0.9))+
  ylim(0,20)+
  annotate("text", x=5, y=18, label = "*")+
  annotate("text", x=4, y=15.8, label = "*")+
  labs(title="Denitrification")+
  xlab("Treatment")+
  ylab(expression('Denitrification Potential Rate (nmol hr'^"-1" ~ 'g'^"-1"*')'))
```

SUPPLEMENTARY REFERENCES

1. La Guardia, M. J., Hale, R. C. & Newman, B. Brominated flame-retardants in sub-saharan Africa: Burdens in inland and coastal sediments in the eThekwini metropolitan municipality, South Africa. *Environ. Sci. Technol.* **47**, 9643–9650 (2013).
